# Relationship between Urinary Level of Phytate and Valvular Calcification in an Elderly Population: A Cross-Sectional Study

**DOI:** 10.1371/journal.pone.0136560

**Published:** 2015-08-31

**Authors:** Carlos Fernández-Palomeque, Andres Grau, Joan Perelló, Pilar Sanchis, Bernat Isern, Rafel M. Prieto, Antonia Costa-Bauzá, Onofre J. Caldés, Oriol Bonnin, Ana Garcia-Raja, Armando Bethencourt, Felix Grases

**Affiliations:** 1 IdISPa, Hospital Universitario Son Espases, 07120, Palma of Mallorca, Spain; 2 Laboratory of Renal Lithiasis Research, Institute of Health Sciences Research (IUNICS- IdISPa), University of Balearic Islands, 07122, Palma of Mallorca, Spain; 3 CIBERobn (CB06/03), Instituto de Salud Carlos III, 28029, Madrid, Spain; Scuola Superiore Sant'Anna, ITALY

## Abstract

Pathological calcification generally consists of the formation of solid deposits of hydroxyapatite (calcium phosphate) in soft tissues. Supersaturation is the thermodynamic driving force for crystallization, so it is believed that higher blood levels of calcium and phosphate increase the risk of cardiovascular calcification. However several factors can promote or inhibit the natural process of pathological calcification. This cross-sectional study evaluated the relationship between physiological levels of urinary phytate and heart valve calcification in a population of elderly out subjects. A population of 188 elderly subjects (mean age: 68 years) was studied. Valve calcification was measured by echocardiography. Phytate determination was performed from a urine sample and data on blood chemistry, end-systolic volume, concomitant diseases, cardiovascular risk factors, medication usage and food were obtained. The study population was classified in three tertiles according to level of urinary phytate: low (<0.610 μM), intermediate (0.61–1.21 μM), and high (>1.21 μM). Subjects with higher levels of urinary phytate had less mitral annulus calcification and were less likely to have diabetes and hypercholesterolemia. In the multivariate analysis, age, serum phosphorous, leukocytes total count and urinary phytate excretion appeared as independent factors predictive of presence of mitral annulus calcification. There was an inverse correlation between urinary phytate content and mitral annulus calcification in our population of elderly out subjects. These results suggest that consumption of phytate-rich foods may help to prevent cardiovascular calcification evolution.

## Introduction

Numerous mechanisms regulate calcium levels in the body, and blood levels of calcium in healthy individuals usually occur within a narrow range. Calcium absorption from the gut, elimination through the kidneys, and deposition into bones all affect the body’s level of calcium [1]. Deposition of solid can occur in a controlled manner, such as during teeth or bone formation, or can be associated with pathological processes such as the formation of dental calculi, dental tartar, kidney stones, chondrocalcinosis, calcinosis cutis, and cardiovascular calcification (CVC). Pathological calcification generally consists of the formation of solid deposits of hydroxyapatite (calcium phosphate) in soft tissues. Other solid calcium salts occur in renal lithiasis (calcium oxalate) and chondrocalcinosis (calcium pyrophosphate).

CVC is a pathological form of soft tissue calcification. Supersaturation is the thermodynamic driving force for crystallization, so it is believed that higher blood levels of calcium and phosphate increase the risk of CVC. However several factors can promote or inhibit the natural process of CVC; vitamin D, lipids, and inflammatorycytokines promote calcification, whereas fetuin-A, pyrophosphate, vitamin K, osteopontin, and matrix Gla protein inhibit CVC [2].Additional factors, including aging and renal insufficiency, can promote CVC [3]. The extent of CVC in subjects at 90 years old is 30-fold equal or greater than in their twenties [4], and dialysis subjects have calcification scores 2 to 5-fold greater than age matched individuals with normal renal function and angiographically proven coronary artery disease [5]. The extent and rate of progression of CVC are strong predictors of cardiovascular events and mortality in the general population, the elderly, subjects with diabetes, and subjects with chronic kidney disease (CKD) who are undergoing dialysis [6–8]. A study of 4 ethnic groups indicated that CVC was a stronger predictor of cardiovascular risk than other classical risk factors such as hypertension and elevated cholesterol [9].

CVC may be classified as intimal or medial, depending on its location in the vessel. Medial CVC is more common in subjects with CKD or diabetes [10], and involves the differentiation of vascular smooth muscle cells into osteoblast-like cells [11]. Intimal CVC seems to be related to aging and is initiated by endothelial injury due to mechanical stress [12], culminating in the formation of atherosclerotic plaque. Early valve lesions seem to be similar to the process of atherosclerosis [13]. Valve calcifications are commonly identified by echocardiography and are associated with stroke and cerebral infarction [14].

Phytate (myo-inositol hexaphosphate) is a naturally occurring substance that the FDA classifies as GRAS (Generally Recognized As Safe). This substance is a powerful inhibitor of crystallization that can block the formation and growth of hydroxyapatite deposits. Previous research indicated that phytate can inhibit the formation of kidney stones [15], sialolithiasis [16], dental tartar [17], and CVC [18]. In this paper, we present a cross-sectional study to describe the relationship between physiological levels of urinary phytate and valve calcification in a population of elderly outpatients.

## Materials and Methods

### Ethics Statement

The study adhered to the Declaration of Helsinki. The Ethics Committee from the Balearic Islands approved the study protocol (Protocol IB 459/05 PI), and all subjects gave their written informed consent.

### Study population

The study sample consisted of 188 consecutive out patients referred by cardiologists to the Echocardiography Laboratory of the Cardiology Department of Son Dureta Hospital (Palma de Mallorca, Spain). The study sample was classified according urinary phytate concentration tertiles. All individuals had unrestricted diets at the time of urine collection. Subjects with chronic kidney disease, end-stage renal disease, a prosthetic valve, aortic or mitral stenosis, or a terminal disease were excluded.

### Measurement of urinary phytate

Phytate determination was performed from a urine sample collected 2 h after the first urination of the morning. Five millilitres of fresh urine (acidified 1:1 with HCl to pH 3–4) was transferred to a column containing 0.2 g of anion-exchange resin (inner diameter: 4 mm), and the first eluate was discarded. The column was then washed with 50 mL of 50 mM HCl, and the second eluate was also discarded. Then, the column was washed with 3 mL of 2 M HNO3. Phytate was determined by direct phosphorus analysis of this last eluate using inductively coupled plasma atomic emission spectroscopy (ICP-AES). Taking into account the sample treatment performed, the lower limit of detection of phytate was 64 μg/L, while the limit of quantification was 213 μg/L. The working linear range used was 0–7 mg/L phytate. The relative standard deviation (R.S.D.) corresponding to five measurements of 1.35 mg/L phytate was 2.4%, the accuracy of the method in spiked samples gave a recovery of 97–105%. [19].

### Measurement of cardiovascular calcification

Transthoracic echocardiography was performed with an echocardiograph (General Electric System Five, GE Healthcare, Buckinghamshire UK). The parasternal long axis, valvular and left ventricular short axis, apical four chamber, two chamber, and long axis projections were obtained. Left atrial volume and diameter and left ventricular end-diastolic and end-systolic diameters were measured. Hemodynamic parameters were obtained with pulsatile and continuous wave Doppler measurement. A mitral annulus calcification (MAC) was defined by the following features on cross-sectional echocardiography in the parasternal window: focal increased echogenicity at the base of the posterior leaflet when visualized in parasternal long axis, short axis, and four chamber views. Aortic valve calcification (AVC) was defined by the presence of irregular cusp thickening and focal hyperechogenicity at the base in the respective projections. The extent of valve calcification was estimated by the Rosenheck score [20], a semi-quantitative method, and ranged from 1 (no calcium) to 4 (severe valve calcification).

### Concomitant diseases, co-medications and blood chemistry

Diseases known as risk factors for atherosclerosis were recorded from each medical history. This includes diabetes, high blood pressure, high plasma cholesterol levels, CKD, renal lithiasis, previous cerebrovascular disease, previous myocardial infarction, vascular disease, arthropathy, osteoporosis, gout, any type of cancer, colon cancer, obesity, and smoking. Use of the following medications was also recorded: acetylsalicylic acid, ticlopidine, statins, fibrate, ezetimibe, angiotensin converting enzyme (ACE) inhibitors, angiotensin receptor blockers (ARBs), diuretics, beta blockers, allopurinol, oral antidiabetics, insulin, calcium antagonists, calcitonin, oral calcium, and thyroid hormone.

Blood samples were collected from all subjects for measurement of the following parameters: calcium, chloride, phosphorus, magnesium, potassium, sodium, creatinine, glucose, intact PTH (iPTH), urea, alkaline phosphatase (ALP), alanine aminotransferase (ALT), aspartate aminotransferase (AST), total bilirubin, uric acid, triglycerides, total cholesterol, high-density lipoprotein (HDL), low-density lipoprotein cholesterol (LDL-C), total protein, albumin, fibrinogen, homocysteine, lipoprotein A (LPA), gamma glutamyl transferase (GGT), haemoglobin, haematocrit, erythrocytes, leukocytes, and the percentage of leucocytes as monocytes, lymphocytes, eosinophils, basophils, and neutrophils. End-systolic volume was also measured by 2-dimensional echocardiography.

### Statistical analysis

Continuous variables are expressed as mean ± standard deviation and categorical variables are expressed as total number (percentage). All continuous variables were checked with normality plots and tests to show their distributions. Continuous variables with normal distributions were compared using one-way analysis of variance (ANOVA) and/or the t test for independent samples. Continuous variables with abnormal distributions were compared using Kruskal-Wallis one-way analysis of variance by ranks and/or Mann-Whitney U tests. For categorical variables, the chi-square test and/or Fisher’s exact test were used.

Univariate and multivariate binary logistic regressions were used to identify risk factors associated with presence of MAC. Multivariate analysis was performed using the stepwise backward method for all models.

A two-tailed *p*-value less than 0.05 was considered statistically significant, unless stated otherwise for multiple comparison. The Bonferroni correction was used to account for multiple comparisons. Within each set of analysis, the significance level (α) used in a given set of tests was equal to 0.05 divided by the number of tests performed in that set.

The statistical package SPSS (Statistical Package for the Social Sciences, version 17.0, SSPS Inc, Chicago, Ill, USA) was used for statistical analyses.

## Results

We studied a population of 188 elderly subjects from a single institution in Spain (mean age: 68± 11 years; 103 males and 85 females).

### Urinary phytate levels, cardiovascular calcification and risk factors

All subjects were classified into their urinary phytate tertiles: low (<0.61 μM), intermediate (0.61–1.21 μM), and high (>1.21 μM). Table 1 shows the age, BMI, sex and cardiovascular risk factors associated with baseline characteristics of subjects according to urinary phytate tertiles. As can be seen, participants in higher phytate tertile were younger compared to lower phytate tertiles (70.5 ± 9.2; 68.9 ± 11.4 and 65.0 ± 11.8 years; *p* = 0.017). Furthermore, the prevalence of diabetes type II is significantly higher at lower phytate tertiles (36.5%, 29.0% and 17.5%; *p* = 0.048).

**Table 1:** Age, BMI, sex and cardiovascular risk factors associated to baseline characteristics of subjects according to urinary phytate level tertiles (low, intermediate and high).

	Urinary phytate levels (μM)																p-value
	Low				Intermediate				High				All subjects				
	T1: < 0.61				T2: 0.61–1.21				T3: > 1.21								
	(n = 63)				(n = 62)				(n = 63)				(n = 188)				
Age (years)	70.5	±	9.2		68.9	±	11.4		65.0	±	11.8	a	68.1	±	11.0		0.017
BMI (kg/m^2^)	27.7	±	5.0		27.7	±	4.7		27.9	±	5.7		27.8	±	5.1		0.960
Sex (male)	32	(	50.8%	)	38	(	61.3%	)	33	(	52.4%	)	103	(	54.8%	)	0.447
Diabetes	23	(	36.5%	)	18	(	29.0%	)	11	(	17.5%	)a	52	(	27.7%	)	0.048
Hypertension	36	(	57.1%	)	37	(	59.7%	)	33	(	52.4%	)	106	(	56.4%	)	0.705
Hypercholesterolemia	35	(	55.6%	)	26	(	41.9%	)	23	(	36.5%	)	84	(	44.7%	)	0.086
Chronic kidney disease	12	(	19.0%	)	5	(	8.1%	)	8	(	12.7%	)	25	(	13.3%	)	0.192
Renal lithiasis	7	(	11.1%	)	11	(	17.7%	)	15	(	23.8%	)	33	(	17.6%	)	0.173
Cerebrovascular disease	11	(	17.5%	)	10	(	16.1%	)	8	(	12.7%	)	29	(	15.4%	)	0.747
Myocardial infarction	9	(	14.3%	)	12	(	19.4%	)	7	(	11.1%	)	28	(	14.9%	)	0.427
Peripheral vascular disease	6	(	9.5%	)	2	(	3.2%	)	4	(	6.3%	)	12	(	6.4%	)	0.354
Arthrosis	31	(	49.2%	)	30	(	48.4%	)	27	(	42.9%	)	88	(	46.8%	)	0.740
Osteoporosis	10	(	15.9%	)	7	(	11.3%	)	6	(	9.5%	)	23	(	12.2%	)	0.533
Gout	7	(	11.1%	)	10	(	16.1%	)	6	(	9.5%	)	23	(	12.2%	)	0.501
Colon cancer	1	(	1.6%	)	0	(	0.0%	)	1	(	1.6%	)	2	(	1.1%	)	0.608
Cancer	8	(	12.7%	)	3	(	4.8%	)	6	(	9.5%	)	17	(	9.0%	)	0.305
Physical exercise	17	(	27.0%	)	20	(	32.3%	)	18	(	28.6%	)	55	(	29.3%	)	0.802
Smoking (currently or past)	20	(	31.7%	)	15	(	24.2%	)	18	(	28.6%	)	53	(	28.2%	)	0.642
Alcohol (currently or past)	2	(	3.2%	)	2	(	3.2%	)	3	(	4.8%	)	7	(	3.7%	)	0.732

Statistics: Continuous variables are expressed as mean ± standard deviation and categorical variables are expressed as total number (percentage). Continuous variables were compared using one-way analysis of variance (ANOVA) and t-test for independent samples. Continuous variables with abnormal distributions were compared using the Kruskal-Wallis one-way analysis of variance by ranks and Mann-Whitney U test. For categorical variables, the chi-square test and Fisher’s exact test were used. The Bonferroni correction was used to account for multiple comparisons. The p-values correspond to the analysis of variance or chi-square test. a: p<0.05/3 *vs*. low group for the *post-hoc* tests.

**Fig 1:**
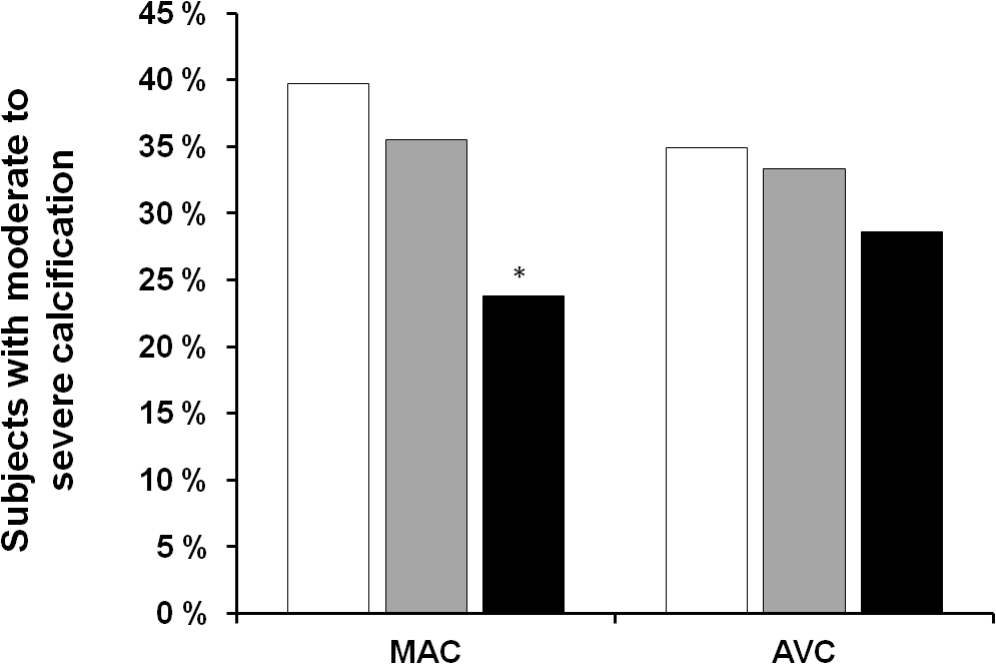
Percentage of subjects with moderate to severe mitral annulus calcification (MAC) and aortic valve calcification (AV) according to urinary phytate level tertiles (Low, <0.61μM, white bars; Intermediate, 0.61–1.21 μM, grey bars; High, >1.21 μM, black bars). Statistics. Values are expressed as percentage. Comparisons between groups were performed using chi-square test and Fisher’s exact test. * *p* < 0.05/3 *vs*. low group.

Analysis of cardiovascular calcification indicated a trend for decreased aortic valve calcification as phytate urinary concentration increased (Fig 1). In particular, moderate to severe aortic valve calcification was present in 34.9% of the low tertile group, 33.3% of the intermediate tertile group, and 28.6% of the high tertile group (*p* = 0.804). However, there was a statistically significant difference in mitral annulus calcification and urinary level of phytate between the low and high phytate groups (39.7% *vs*. 23.8%, *p* = 0.015).

Table 2 shows the medication usage according to urinary phytate level tertiles, for which no statistically significant differences were observed. Table 3 shows the blood chemistry and end-systolic volume results of the 3 groups of subjects. These groups had statistically significant differences in serum sodium, creatinine, haemoglobin, urea, total cholesterol, LDL-C, erythrocyte count, and haematocrit.

**Table 2:** Co-medication usage of subjects according to urinary phytate level tertiles (low, intermediate and high).

	Urinary phytate levels (μM)																p-value
	Low				Intermediate				High				Allsubjects				
	T1: < 0.61				T2: 0.61–1.21				T3: > 1.21								
	(n = 63)				(n = 62)				(n = 63)				(n = 188)				
Acetylsalicylic acid	19	(	30.2%	)	16	(	25.8%	)	20	(	31.7%	)	55	(	29.3%	)	0.752
Ticlopidine	4	(	6.3%	)	7	(	11.3%	)	5	(	7.9%	)	16	(	8.5%	)	0.601
Statins	25	(	39.7%	)	21	(	33.9%	)	19	(	30.2%	)	65	(	34.6%	)	0.526
Fibrate	1	(	1.6%	)	2	(	3.2%	)	3	(	4.8%	)	6	(	3.2%	)	0.598
Ezetimibe	0	(	0.0%	)	0	(	0.0%	)	1	(	1.6%	)	1	(	0.5%	)	0.369
ACE inhibitors	19	(	30.2%	)	20	(	32.3%	)	19	(	30.2%	)	58	(	30.9%	)	0.958
ARBs	10	(	15.9%	)	12	(	19.4%	)	8	(	12.7%	)	30	(	16.0%	)	0.597
Proximal diuretics	6	(	9.5%	)	6	(	9.7%	)	3	(	4.8%	)	15	(	8.0%	)	0.513
Distal diuretics	16	(	25.4%	)	14	(	22.6%	)	16	(	25.4%	)	46	(	24.5%	)	0.915
Beta blockers	17	(	27.0%	)	19	(	30.6%	)	17	(	27.0%	)	53	(	28.2%	)	0.871
Oral antidiabetics	14	(	22.2%	)	9	(	14.5%	)	5	(	7.9%	)	28	(	14.9%	)	0.079
Insulin	11	(	17.5%	)	8	(	12.9%	)	3	(	4.8%	)	22	(	11.7%	)	0.080
Calcium antagonist	16	(	25.4%	)	12	(	19.4%	)	6	(	9.5%	)	34	(	18.1%	)	0.065
Calcitonin	3	(	4.8%	)	1	(	1.6%	)	1	(	1.6%	)	5	(	2.7%	)	0.445

Statistics: Variables are expressed as total number (percentage). Comparisons between groups were performed by the chi-square test.

**Table 3:** Blood chemistry and end-systolic volume in subjects according to urinary phytate level tertiles (low, intermediate and high).

	Urinary phytate levels (μM)																
	Low				Intermediate				High				All subjects				p-value
	T1: < 0.61				T2: 0.61–1.21				T3: > 1.21								
	(n = 63)				(n = 62)				(n = 63)				(n = 188)				
Calcium (mg/dL)	9.00	±	0.70		8.92	±	0.38		9.03	±	0.54		8.98	±	0.55		0.533
Chloride (mEq/L)	101	±	4		102	±	4		102	±	4		102	±	4		0.163
Phosphorous (mg/dL)	3.29	±	0.81		3.18	±	0.62		3.29	±	0.86		3.25	±	0.77		0.713
Magnesium (mEq/dL)	1.56	±	0.50		1.62	±	0.52		1.68	±	0.47		1.62	±	0.50		0.385
Potassium (mEq/L)	4.08	±	0.42		3.98	±	0.50		4.02	±	0.43		4.03	±	0.45		0.418
Sodium (mEq/L)	141.0	±	2.5		142.1	±	2.7	a	141.6	±	2.9		141.5	±	2.7		0.049
Creatinine (mg/dL)	0.70	±	0.87		0.44	±	0.50		0.39	±	0.66		0.51	±	0.71		0.045
Glucose (mg/dL)	120	±	37		113	±	33		111	±	34		115	±	34		0.105
Haemoglobin (g/dL)	12.3	±	1.9		13.3	±	4.4		13.1	±	2.1	a	12.9	±	3.0		0.040
Urea(mg/dL)	58	±	31		46	±	18	a	48	±	38		50	±	30		0.021
Uric acid (mg/dL)	6.0	±	2.2		5.7	±	2.1		5.5	±	1.7		5.7	±	2.0		0.402
Total cholesterol (mg/dL)	182	±	42		193	±	34		199	±	53		191	±	44		0.041
HDL (mg/dL)	54	±	16		53	±	15		55	±	17		54	±	16		0.862
LDL-C (mg/dL)	100	±	37		110	±	33		114	±	50	a	108	±	41		0.049
Triglycerides (mg/dL)	137	±	66		129	±	81		131	±	63		133	±	70		0.330
Total protein (g/L)	70	±	8		71	±	6		72	±	5		71	±	6		0.589
Albumin (g/L)	41.2	±	5.3		42.7	±	4.0		42.2	±	3.47		42.0	±	4.3		0.191
ALP (U/L)	81	±	26		74	±	24		76	±	31		77	±	27		0.174
ALT (U/L)	27	±	24		20	±	10		25	±	23		24	±	20		0.285
AST (U/L)	23	±	11		20	±	7		21	±	16		22	±	12		0.058
Gamma glutamyltransferase (U/L)	46	±	56		30	±	24		33	±	30		37	±	40		0.610
Haematocrit (%)	37.7	±	5.4		39.3	±	4.9		39.9	±	5.5	a	39.0	±	5.3		0.046
Erythrocytes (x10^6^ /μL)	3.87	±	0.75		4.13	±	0.59		4.19	±	0.69	a	4.06	±	0.69		0.033
Leukocytes (x10^3^/μL)	9	±	16		7	±	2		7	±	2		8	±	10		0.854
Lymphocytes (%)	29	±	11		28	±	8		29	±	9		29	±	9		0.850
Neutrophils (%)	59	±	12		60	±	10		58	±	11		59	±	11		0.867
Basophils (%)	0.54	±	0.50		0.52	±	0.27		0.53	±	0.28		0.53	±	0.37		0.643
Eosinophils (%)	2.5	±	1.9		2.5	±	1.8		2.2	±	1.4		2.4	±	1.7		0.574
Monocytes (%)	7.7	±	1.9		7.5	±	1.9		7.6	±	2.1		7.6	±	2.0		0.830
Neutrophil/Lymphocyte ratio	2.6	±	1.7		2.6	±	1.9		2.4	±	2.0		2.5	±	1.9		0.871
iPTH (pg/mL)	62	±	45		57	±	32		56	±	35		59	±	38		0.823
Fibrinogen (mg/dL)	410	±	128		393	±	109		407	±	142		403	±	127		0.661
Homocysteine (μM)	10.3	±	6.3		11.8	±	14.2		11.7	±	15.5		11.3	±	12.5		0.887
Lipoprotein A(mg/dL)	60	±	57		45	±	32		49	±	46		51	±	46		0.609
End-systolic volume (mL)	29	±	31		19	±	17		23	±	29		24	±	26		0.094

Statistics: Continuous variables are expressed as mean ± standard deviation and categorical variables are expressed as total number (percentage). Continuous variables with normal distributions were compared using one-way analysis of variance (ANOVA) and t-test for independent samples. Continuous variables with abnormal distributions were compared using the Kruskal-Wallis one-way analysis of variance by ranks and Mann-Whitney U test. For categorical variables, the chi-square test was used. The Bonferroni correction was used to account for multiple comparisons. The p-values correspond to the analysis of variance or chi-square test. a: p<0.05/3 *vs*. low group for the *post-hoc* tests.

### Risk factors associated with the presence of MAC

Subjects were classified into 2 categories according to the presence or absence of MAC. Of whole sample, 32.1% presented MAC.

Demographics and risk factors, blood chemistry end-systolic volume of both groups are shown in Table 4. Subjects were older (73.1 ± 8.2 *vs*. 66.0 ± 11.4 years; p < 0.001) and the prevalence of diabetes type II was higher in subjects with MAC than in those without MAC (37.1% vs. 23.0%, p = 0.039). In relation to blood chemistry, serum phosphorous, glucose, leukocytes, neutrophils and neutrophil/lymphocyte ratio were significantly higher in subjects with MAC in comparison to those without MAC, whereas haematocrit, erythrocytes and lymphocytes were significantly lower in MAC group compared to without MAC group. Also end-systolic volume were significantly lower in MAC group compared to without MAC group.

**Table 4:** Demographics and risk factors, blood chemistry and end-systolic volume of the study population according to presence or absence of mitral valve calcification (MAC and No-MAC).

	MAC				No-MAC				p-value
	(n = 62)				(n = 126)				
**Demographics and risk factors**									
Age (years)	73.1	±	8.2		66.0	±	11.4		<0.001
BMI (kg/m^2^)	28.0	±	5.1		27.9	±	5.1		0.578
Sex (male)	30	(	48.4%	)	73	(	57.9%	)	0.275
Hypertension	41	(	66.1%	)	65	(	51.6%	)	0.063
Diabetes type II	23	(	37.1%	)	29	(	23.0%	)	0.039
Hypercholesterolemia	29	(	46.8%	)	55	(	43.7%	)	0.756
Chronic kidney disease	11	(	17.7%	)	14	(	11.1%	)	0.254
Cerebrovascular disease	8	(	12.9%	)	21	(	16.7%	)	0.668
Myocardial infarction	12	(	19.4%	)	16	(	12.7%	)	0.276
Peripheral vascular disease	4	(	6.5%	)	8	(	6.3%	)	1.000
Smoking (currently or past)	18	(	29.0%	)	35	(	27.8%	)	1.000
Physical exercise	20	(	32.3%	)	35	(	27.8%	)	0.609
Alcohol (currently or past)	9	(	14.5%	)	30	(	23.8%	)	0.248
**Blood chemistry and end-systolic volume**									
Calcium (mg/dL)	9.0	**±**	0.6		9.0	**±**	0.6		0.783
Phosphorous (mg/dL)	3.5	**±**	0.8		3.1	**±**	0.7		0.002
Magnesium (mEq/dL)	1.6	**±**	0.5		1.6	**±**	0.5		0.800
Creatinine (mg/dL)	0.6	**±**	0.8		0.5	**±**	0.7		0.165
Glucose (mg/dL)	124	**±**	42		111	**±**	31		0.046
Uric acid (mg/dL)	6	**±**	2		5	**±**	2		0.064
Total cholesterol (mg/dL)	182	**±**	33		194	**±**	46		0.071
HDL (mg/dL)	52	**±**	15		55	**±**	16		0.303
LDL-C (mg/dL)	101	**±**	32		111	**±**	44		0.220
Triglycerides (mg/dL)	132	**±**	72		135	**±**	73		0.843
Haematocrit (%)	37	±	6		40	±	5		0.001
Erythrocytes (x10^6^/μl)	3.9	±	0.7		4.2	±	0.7		0.006
Leukocytes (x10^3^/μL)	9.6	**±**	6.3		6.7	**±**	2.2		0.019
Lymphocytes (%)	28	±	11		29	±	8		0.029
Neutrophils (%)	61	**±**	12		58	**±**	10		0.037
Eosinophils (%)	2.3	±	1.6		2.5	±	1.8		0.641
Basophils (%)	0.55	±	0.34		0.51	±	0.42		0.076
Monocytes (%)	7.6	±	1.9		7.6	±	2.1		0.836
Neutrophil/Lymphocyte ratio	2.7	**±**	1.6		2.4	**±**	1.9		0.022
iPTH (pg/mL)	56	**±**	35		60	**±**	39		0.728
Fibrinogen (mg/dL)	416	**±**	138		396	**±**	120		0.186
Homocystein (μM)	11.5	**±**	8.5		11.1	**±**	9.1		0.341
Lipoprotein A (mg/dL)	48	**±**	37		59	**±**	60		0.737
End-systolic volume (mL)	20	±	22		30	±	31		0.011

Statistics. Values are expressed as mean ± SD or frequency (percentage). Continuous variables with normal distributions were compared using t test for independent samples. Continuous variables with abnormal distributions were compared using Mann-Whitney U test. For categorical variables, Fisher’s exact test was used.

**Fig 2:**
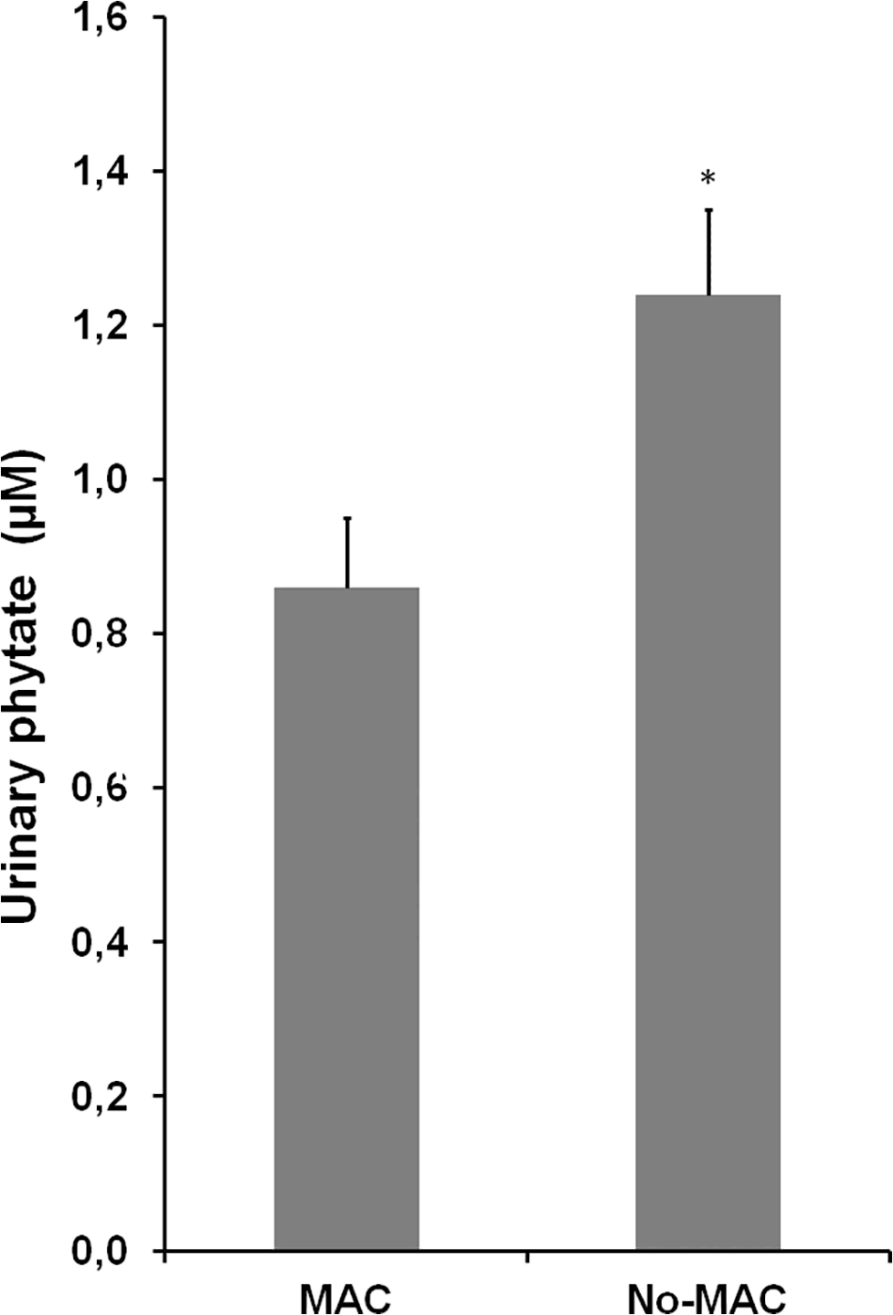
Urinary phytate levels for subjects with mitral annulus calcification (MAC) and without MAC (No MAC). Statistics. Values are expressed as mean ± SE. Comparisons between groups were performed using Mann-Whitney U test. * *p* < 0.05 *vs*. No MAC.

Regarding urinary phytate levels, subjects without MAC had significantly higher levels of urinary phytate than subjects with MAC (Fig 2).

As can be seen in Table 5, on univariate binary logistic regression, MAC was significantly associated with age, diabetes, serum glucose, urinary phytate excretion, leukocytes, and phosphorous levels. We also performed multiple binary logistic regression analysis to identify factors that were independently associated with the presence of MAC. All the previously listed factors were included initially in the model before stepwise and backward elimination. Diabetes was significantly correlated with phytate (r = -0.160; *p* = 0.028) and serum phosphorus (r = 0.185, *p* = 0.012), so it was eliminated from the final model. In the final multivariate model adjusted by age, leukocytes, serum phosphorous and urinary phytate excretion were independent associated factors to presence of MAC.

**Table 5:** Binary logistic regression of risk factors associated with presence of mitral annulus calcification (MAC).

	Unadjusted Odds Ratio (O.R.)	(95% C.I.for O.R.)				p-value		Adjusted Odds Ratio (O.R.)	(95% C.I.for O.R.)				p-value	

Age (years)	1.076	(	1.038	-	1.115	)	<0.0001	1.083	(	1.041	-	1.128	)	<0.0001
Phytate (μM)	0.599	(	0.387	-	0.929	)	0.022	0.640	(	0.414	-	0.990	)	0.045
Leukocytes (x10^3^/μL)	1.138	(	1.009	-	1.284	)	0.035	1.152	(	1.005	-	1.320	)	0.042
Phosphorous (mg/dL)	1.881	(	1.224	-	2.892	)	0.004	1.970	(	1.199	-	3.239	)	0.007
Diabetes (yes *vs*. no)	2.178	(	1.127	-	4.208	)	0.021							
Hypertension (yes vs. no)	1.832	(	0.974	-	3.445	)	0.060							
Glucose (mg/dL)	1.009	(	1.000	-	1.018	)	0.052							
Total cholesterol (mg/dL)	1.008	(	0.999	-	1.016	)	0.068							
Neutrophil/Lymphocyte ratio	1.079	(	0.922	-	1.262	)	0.342							

Statistics: Univariate and multivariate binary logistic regressions were used to identify risk factors associated with presence of MAC. Multivariate analysis was performed using the stepwise backward method.

## Discussion

In this study we demonstrated a significantly inverse correlation between urinary phytate level and MAC. Phytate is a naturally occurring component in the diet and the FDA classifies it as GRAS. It is a highly polar substance, and its oral bioavailability is very low because of limited absorption [15], partly due to metabolic dephosphorylation during the first hepatic pass [21]. Previous research indicated that a maximum of 2% oral bioavailability [22], and that increasing oral intake had little effect on maximum plasma values (0.3–0.4 μM) [23]. In several aspects, phytate resembles some bisphosphonates, which also have high polarity and poor absorption [24].

However, bisphosphonates are non-hydrolysable pyrophosphate derivatives that have long physiological half-lives. Other research indicated that urinary phytate levels reflect the phytate status of an organism, and that there is a good correlation between plasma and urinary levels of phytate [25].

Phytate in humans and other mammals occurs in intracellular and extracellular compartments. Intracellular phytate (concentration range: 10–100 μM) occurs following sequential phosphorylation of lower phosphoinositides [26], but its physiological role remains unclear. There is apparently no exchange of phytate between the intracellular and extracellular compartments [27]. Extracellular (blood) phytate is usually below 0.4 μM and its concentration depends exclusively on dietary intake [28].

The major sources of dietary phytate are whole grains, legumes, beans, and vegetable seeds [15]. It has been estimated that the daily intake of phytate on the basis of western style diets varies from ~ 0.3–2.6 g and in a global range from 0.180–4.569 g [15], strongly depending on the diet selected; low in normal western diets and high in vegetarian diets. Phytate is predominantly present in unprocessed food, but can be degraded during processing. Under heat treatment up to ~ 100°C (home cooking, roasting, pressure cooking etc.) phytate is quite stable [15]. For decades phytate has been regarded as an antinutrient, it may inhibit the absorption of some essential trace elements and minerals, although only under mineral malnutrition would lead to calcium, iron and zinc deficiencies [15]. The trace elements and mineral malnutrition is produced under non varied and non balanced dietary conditions. The complex interaction between food compounds makes difficult to accurately determine the exact effects of these relationships, i. e. with carotenoids or vitamin C on mineral bioavailability. In a previous study we found that Mediterranean diet high in whole cereals, legumes and nuts compared to Mediterranean diet low in these phytate-rich foods increased significantly the urinary phytate excretion in humans [29].

These previous studies and the results presented here support the view that dietary consumption of phytate is crucial for maintaining adequate physiological levels of this compound, and that a phytate-rich diet may help to protect against valve calcification. In fact, the reduced phytate consumption in many industrialized countries could be partially responsible for the increasing prevalence of vascular and valve calcification in these countries. The extent to which low phytate consumption is responsible for vascular and valve calcification is unclear, but dietary phytate deserves further attention as a potentially protective factor.

There is a clear tendency for improved cardiovascular health in subjects with higher levels of urinary phytate (Table 3), especially in terms of hypercholesterolemia and diabetes. Although phytate may have a direct effect on vessel and valve calcification because it lowers serum cholesterol [30, 31], the role of lipid deposition in the atherosclerotic process must also be considered. The process of valve calcification starts with a lesion (an endothelial injury) and continues by extracellular lipid accumulation in the subendothelial region, a process exacerbated by high levels of oxidized LDL. Introducing a crystallization inhibitor in this process can block or delay calcification, and phytate is one of the most powerful inhibitors of calcium salt crystallization. *In vitro* studies have shown that phytate inhibits calcium phosphate crystallization [15] and *in vivo* studies have shown it can prevent calcium-related diseases, such as renal stones [15], sialolithiasis [16], dental tartar [17] in humans, and cardiovascular calcification [18] in animal models.

Regarding independent factors associated to MAC, previous studies have reported that age, serum phosphorus levels and leukocytes are strongly associated with vascular calcification [32–37]. Valve calcification is a multifactorial process where calcium/phosphorous saturation and inflammatory/immunological events are considered to play a central role in its initiation and progression [34–37]. Indeed, elevation in serum phosphorous levels are frequently reported in subjects with vascular calcification [34]. In recent years, some studies have indicated that circulation leukocytes [36, 37] and neutrophil/lymphocytes ratio [35] are independent predictors of mortality and cardiovascular events.

Nevertheless, in our study, 67.0% of subjects present no MAC and among these subjects, 28.6% have more than 65 years. These finding are in line with previous observations [38, 39] that indicate that some elderly subjects had no visible calcification, even though some of them were > 80 years of age. All these data suggest that some individuals rarely develop calcification because some biochemical, dietary and/or genetically features protect them from calcification.

According our results, phytate rich-food consumption can be one of these features that protect subjects from calcification. Dietary phytate treatment has demonstrated to reduce drastically age-related aortic calcification in rats [40] and it is possible that these results could be extrapolated to humans.

To the best of our knowledge, the present study is the first prospective observational clinical data to identify a correlation between high urinary level of phytate and low cardiovascular (valve) calcification. These results suggest that increased consumption of phytate rich foods may help to prevent or minimize these dystrophic calcifications.

## Supporting Information

S1 Dataset Dataset. Relationship between Urinary Level of Phytate and Valvular Calcification in an Elderly Population: a Cross-Sectional Study.
(PDF)
