# Supplementary material for: Relationship between Urinary Level of Phytate and Valvular Calcification in an Elderly Population: A Cross-Sectional Study
Source: PLoS One. 2015 Aug 31;10(8):e0136560. doi: 10.1371/journal.pone.0136560 (PMC4554994; doi:10.1371/journal.pone.0136560)
Supplement: S1 Dataset — Relationship between Urinary Level of Phytate and Valvular Calcification in an Elderly Population: a Cross-Sectional Study. (PDF) [file pone.0136560.s001.pdf]

Dataset. Relationship between Urinary Level of Phytate and Valvular Calcification in an Elderly Population: a Cross-Sectional Study

Subject	Urinary volume (mL)	pH	Phytate (mg/L)	SEM	Age (years)	Sex (H:male; M:female)	Height (m)	Weight (kg)	BMI (kg/m2)	Diabetes_1yes_2not	HTA_1yes_2not	HCol_1yes_2not	IRC_1yes_2not	RenalLit_1yes_2not	ACV_1yes_2not	IAM_1yes_2not	EVPer_1yes_2not	Artr_1yes_2not	OPor._1yes_2not	Gout_1yes_2not	C.Colon_1yes_2not	Cancer_1yes_2not	AAS_1yes_2not	Ticlopid._1yes_2not	Estatin_1yes_2not	Fibrat2_1yes_2not
1	102	7	0,0	2	88	M	1,58	52	20	1	1	1	1	2	1	2	2	1	1	2	1	1	1	2	2	2
2	99	6	0,0	2	66	H	1,7	64	22	1	1	2	1	2	1	2	2	1	2	2	2	2	1	2	2	2
3	100	5	0,0	2	72	H	1,69	66	23	1	2	1	2	2	2	2	2	2	2	2	2	2	1	2	1	2
4	61	6	0,0	0	59	H	1,73	86	28	1	2	1	2	2	2	2	2	1	2	2	2	2	2	2	2	2
5	84	5	0,0	1	53	M	1,52	73	31	2	2	1	2	1	2	2	2	1	1	2	2	2	2	2	2	2
6	73	6	0,0	6	55	H	1,75	112	36	2	1	2	2	1	2	2	2	2	2	2	2	2	2	2	2	2
7	102	5	0,0	2	67	H	1,6	94	37	2	1	2	2	2	2	2	2	1	2	2	2	2	2	2	2	2
8	83	7	0,01	2	80	M	1,53	52	22	2	2	2	2	2	2	2	2	2	2	2	2	2	2	2	2	2
9	115	5	0,03	3	72	H	1,63	66	25	2	1	2	1	2	2	1	2	2	2	2	2	2	1	2	2	2
10	128	6	0,04	6	79	M	1,54	56	23	2	2	2	2	2	2	2	2	2	2	2	2	2	2	2	2	2
11	62	7	0,06	6	71	M	1,65	65	23	2	1	2	2	2	2	2	2	1	2	2	2	2	2	2	1	2
12	99	4	0,07	3	71	H	1,73	76	25	2	1	2	1	2	2	1	1	2	2	1	2	2	1	2	2	2
13	70	7	0,07	8	55	H	1,73	79	26	2	2	2	2	2	2	2	2	2	2	2	2	2	2	2	2	2
14	80	5	0,09	6	53	H	1,68	82	29	2	2	2	2	2	2	2	2	2	2	2	2	2	1	2	2	2
15	25	6	0,11	8	62	M	1,55	71	29	1	1	1	2	1	1	1	2	2	2	2	2	2	2	2	1	2
16	80	5	0,12	1	78	M	1,56	67	27	2	1	1	1	1	2	2	2	1	1	2	2	1	2	2	2	2
17	65	5	0,14	3	84	H	1,7	75	25	2	2	2	2	1	2	2	2	1	1	2	2	2	2	2	2	2
18	100	6	0,15	4	81	M	1,63	58	22	2	2	2	2	2	2	2	2	2	2	2	2	2	2	2	2	2
19	128	6	0,15	8	76	M	1,6	75	29	1	1	1	1	2	1	1	2	2	2	2	2	2	2	2	1	2
20	91	5	0,15	2	76	H	1,52	70	30	2	1	1	2	2	2	2	2	1	1	2	2	2	2	2	2	2
21	103	5	0,15	2	74	H	1,75	101	32	1	2	1	2	2	2	2	2	2	2	2	2	2	2	2	1	2
22	76	5	0,15	8	68	M	1,59	105	41	1	1	1	2	2	2	2	2	1	2	1	2	2	2	2	2	2
23	52	6	0,16	2	73	H	1,63	83	31	1	1	1	2	2	1	1	2	1	2	1	2	2	1	2	1	2
24	50	5	0,18	5	75	H	1,64	64	23	2	1	2	2	2	2	2	2	1	2	2	2	2	1	2	1	2
25	93	7	0,18	0	73	M	1,42	57	28	2	1	1	2	2	2	2	2	1	1	2	2	2	1	2	1	2
26	102	6	0,18	5	75	M	1,62	92	35	1	1	1	2	2	2	2	2	1	1	2	2	2	2	2	2	2
27	42	5	0,19	1	83	H	1,64	82	30	2	1	2	1	2	2	2	2	2	2	2	2	2	2	2	2	2
28	95	5	0,19	3	70	H	1,77	99	31	2	1	1	2	2	2	2	1	2	2	2	2	1	2	2	1	2
29	60	5	0,2	4	69	H	1,49	42	19	2	2	2	2	2	2	2	2	1	1	2	2	1	2	2	2	2
30	77	6	0,21	2	64	M	1,61	56	21	2	2	1	2	2	2	2	2	1	1	2	2	2	2	2	2	2
31	81	5	0,21	7	59	H	1,6	55	21	1	1	2	1	2	2	2	1	2	2	2	2	2	1	2	1	2
32	110	5	0,22	4	72	H	1,66	80	29	2	1	1	2	2	2	2	1	1	2	2	2	2	1	2	1	2
33	92	6	0,22	1	80	M	1,53	80	34	1	1	1	2	2	2	2	2	1	2	2	2	2	2	2	1	2
34	108	6	0,23	1	85	H	1,75	82	26	1	2	1	2	2	2	2	2	2	2	2	2	2	2	2	2	1
35	115	6	0,23	5	59	M	1,52	62	26	1	1	1	2	2	2	1	2	1	2	2	2	2	1	1	2	2
36	18	7	0,24	2	70	M	1,54	60	25	2	1	1	2	2	2	2	2	2	2	2	2	2	2	2	1	2
37	87	5	0,25	2	76	H	1,63	77	28	2	1	1	2	2	2	2	2	1	2	2	2	1	2	2	1	2
38	97	5	0,25	1	45	H	1,63	110	41	2	2	1	2	2	2	2	2	2	2	2	2	2	2	2	1	2
39	105	6	0,26	3	69	H	1,72	79	26	1	1	2	2	1	2	2	2	2	2	2	2	1	2	2	2	2
40	102	5	0,26	1	69	H	1,62	84	32	1	1	1	1	2	2	2	2	2	2	1	2	2	1	2	1	2
41	60	5	0,28	3	82	M	1,63	58	22	2	2	1	2	2	2	2	2	2	2	2	2	2	2	1	1	2
42	100	5	0,3	3	77	M	1,55	55	23	2	2	1	2	2	1	2	2	1	2	2	2	1	1	2	1	2
43	60	5	0,3	4	75	M	1,55	62	25	1	1	2	2	1	1	2	2	2	2	2	2	2	2	2	2	2
44	86	6	0,31	6	56	H	1,71	82	28	1	2	1	2	2	2	1	2	2	2	2	2	2	1	2	1	2
45	62	5	0,32	2	78	M	1,59	57	22	2	2	2	2	2	2	2	2	2	2	2	2	2	2	2	2	2
46	65	5	0,32	6	50	M	1,58	76	30	2	2	1	2	2	2	2	2	2	2	2	2	2	2	2	1	2
47	56	6	0,32	2	77	M	1,49	71	32	2	1	2	2	2	1	2	2	2	2	2	2	2	2	2	2	2
48	79	6	0,33	1	74	M	1,6	88	34	1	1	2	1	2	2	2	2	1	1	2	2	2	2	2	2	2
49	110	7	0,34	1	68	M	1,66	66	23	2	2	2	2	2	1	2	2	2	2	2	2	2	1	2	2	2
50	55	7	0,34	4	69	M	1,5	65	28	2	2	1	2	2	2	2	2	2	2	2	2	2	2	2	2	2
51	52	5	0,36	3	76	H	1,66	82	29	1	1	2	1	2	2	1	1	1	2	1	2	2	2	2	2	2
52	62	7	0,36	2	78	H	1,54	76	32	2	2	2	2	2	2	2	2	2	2	2	2	2	2	2	2	2
53	21	6	0,37	2	79	M	1,57	74	30	2	2	2	2	2	2	2	2	2	2	2	2	2	2	2	2	2
54	83	5	0,37	2	81	H	1,72	73	24	2	2	1	2	2	2	1	2	2	2	2	2	2	1	2	2	2
55	78	6	0,37	8	66	M	1,56	81	33	1	1	1	2	2	2	2	2	1	2	2	2	2	2	2	1	2
56	68	5	0,38	0	62	M	1,57	56	22	2	1	2	2	2	2	2	2	1	2	2	2	2	1	2	2	2
57	85	6	0,38	1	73	H	1,72	71	24	2	2	1	2	2	1	2	1	1	2	2	2	2	2	1	1	2
58	82	5	0,38	7	62	H	1,56	63	25	2	2	1	2	2	1	2	2	2	2	1	2	2	2	1	1	2
59	141	5	0,38	1	75	H	1,61	65	25	1	1	1	1	2	2	2	2	1	2	1	2	2	2	2	1	2
60	83	5	0,38	2	70	M	1,6	75	29	1	1	2	2	2	2	2	2	1	2	2	2	1	2	2	2	2
61	21	5	0,38	1	73	M	1,55	87	36	2	1	1	2	2	2	2	2	1	2	2	2	2	1	2	2	2
62	59	5	0,39	4	60	H	1,54	59	25	2	1	2	2	2	2	2	2	1	2	2	2	2	2	2	2	2
63	56	5	0,39	8	73	M	1,56	69	28	2	2	1	2	2	2	2	2	1	2	2	2	2	2	2	1	2
64	21	7	0,4	3	82	M	1,56	66	27	1	1	1	2	2	2	1	2	1	2	2	2	2	1	2	2	2
65	52	5	0,41	1	70	H	1,66	72	26	2	2	1	2	2	1	1	2	1	2	2	2	2	2	1	1	2
66	61	5	0,41	4	83	H	1,74	80	26	2	2	2	2	2	2	2	2	1	2	2	2	2	1	2	2	2
67	72	5	0,43	6	52	M	1,59	68	26	2	1	1	1	2	2	2	2	1	1	1	2	2	2	2	2	2
68	71	5	0,43	2	74	M	1,37	78	41	2	2	2	2	1	2	2	2	1	2	2	2	2	2	2	2	2
69	70	5	0,45	2	78	M	1,49	60	27	2	1	1	2	2	2	2	2	1	2	1	2	2	2	2	1	2
70	85	5	0,45	2	58	M	1,57	68	27	2	2	2	2	2	2	2	2	2	2	2	2	2	2	2	2	2
71	41	4	0,45	1	68	H	1,7	80	27	2	1	2	2	2	1	2	2	2	2	2	2	2	2	2	2	2
72	53	5	0,45	4	71	H	1,69	81	28	1	1	2	2	2	2	1	2	2	2	2	2	2	1	1	2	2
73	78	5	0,45	2	69	H	1,69	90	31	1	1	1	2	2	2	2	2	2	2	2	2	2	2	2	2	2
74	88	5	0,46	1	55	H	1,7	92	32	2	1	1	2	2	2	2	2	2	2	1	2	2	2	2	1	2
75	82	6	0,47	6	78	H	1,66	79	28	2	2	2	2	2	1	1	2	1	2	2	2	2	1	2	1	2
76	59	5	0,48	0	76	H	1,67	61	22	2	2	1	2	2	2	2	2	2	2	2	2	1	2	2	2	2
77	62	6	0,48	0	65	H	1,73	77	25	2	2	2	2	1	2	2	2	1	2	2	2	2	2	2	2	2
78	60	4	0,49	8	65	M	1,74	78	25	1	1	1	2	2	1	1	2	1	1	2	2	2	2	1	2	2
79	25	5	0,49	4	76	M	1,49	81	36	1	1	2	2	2	2	2	2	2	2	2	2	2	2	2	2	2
80	103	4	0,51	1	61	H	1,71	76	25	2	1	1	1	2	2	2	2	2	2	1	2	2	2	2	1	2
81	80	5	0,51	3	43	H	1,65	95	35	2	1	1	2	1	2	2	2	2	2	2	2	2	2	2	2	2
82	78	5	0,52	1	79	H	1,7	74	25	2	2	2	2	2	2	2	2	2	2	1	2	2	1	2	2	2
83	94	9	0,52	3	68	H	1,64	68	25	2	2	2	2	2	2	2	2	2	2	2	2	2	2	2	2	2
84	50	6	0,53	2	79	H	1,69	76	26	2	2	2	2	2	1	2	1	2	2	2	2	1	2	2	2	2
85	90	5	0,53	3	68	H	1,64	78	29	2	2	2	2	2	2	1	2	2	2	2	2	2	1	2	2	1
86	81	6	0,54	9	88	H	1,73	72	24	2	1	2	2	2	2	2	2	2	2	2	2	2	2	2	2	2
87	80	5	0,54	0	75	H	1,69	92	32	1	1	1	1	2	2	2	2	2	2	1	2	2	2	2	2	2
88	79	4	0,55	3	59	H	1,79	83	25	2	2	1	2	2	2	2	2	1	2	2	2	2	2	2	1	2
89	21	5	0,55	4	69	M	1,57	74	30	2	1	1	2	2	2	2	2	1	2	2	2	2	2	2	1	2
90	40	6	0,55	7	72	H	1,61	91	35	2	1	1	2	1	2	2	2	1	1	2	2	2	1	1	1	2
91	48	6	0,56	0	35	M	1,6	62	24	2	2	2	2	2	2	2	2	2	2	2	2	2	2	2	2	2
92	83	5	0,56	6	61	H	1,63	84	31	1	2	1	2	2	2	2	2	2	2	1	2	2	2	1	2	2
93	82	5	0,57	2	45	M	1,53	59	25	2	2	2	2	2	2	2	2	2	2	2	2	1	2	2	2	2
94	40	5	0,58	6	73	H	1,7	78	26	2	1	2	2	1	2	1	2	1	2	2	2	2	2	2	2	2
95	23	6	0,58	6	86	M	1,48	63	28	2	2	1	2	2	2	2	2	1	2	2	2	2	2	2	1	2

Dataset. Relationship between Urinary Level of Phytate and Valvular Calcification in an Elderly Population: a Cross-Sectional Study

Subject	Urinary volume (mL)	pH	Phytate (mg/L)	SEM	Age (years)	Sex (H:male; M:female)	Height (m)	Weight (kg)	BMI (kg/m2)	Diabetes_1yes_2not	HTA_1yes_2not	HCol_1yes_2not	IRC_1yes_2not	RenalLit_1yes_2not	ACV_1yes_2not	IAM_1yes_2not	EVPer_1yes_2not	Artr_1yes_2not	OPor._1yes_2not	Gout_1yes_2not	C.Colon_1yes_2not	Cancer_1yes_2not	AAS_1yes_2not	Ticlopid._1yes_2not	Estatin_1yes_2not	Fibrat2_1yes_2not
96	72	5	0,58	2	71	H	1,63	78	29	1	1	1	2	2	1	1	2	1	2	2	2	2	2	2	1	2
97	58	6	0,59	3	83	M	1,53	42	17	2	1	2	2	2	2	2	2	2	2	2	2	2	2	2	2	2
98	80	5	0,59	7	52	M	1,74	75	24	1	2	2	2	2	1	2	2	1	2	2	2	2	2	2	1	2
99	81	4	0,59	2	77	H	1,67	71	25	2	1	2	2	2	2	1	2	2	2	2	2	2	1	2	1	2
100	71	7	0,6	2	77	H	1,78	90	28	2	1	1	2	2	2	2	2	1	2	2	2	2	1	2	1	2
101	91	5	0,61	2	72	H	1,74	78	25	1	2	1	2	2	1	2	2	2	2	2	2	2	1	2	2	2
102	115	5	0,61		75	H	1,64	75	28	2	1	2	2	2	2	2	2	2	2	1	2	2	2	2	2	2
103	16	8	0,62	3	74	M	1,5	58	25	1	1	2	1	2	2	2	2	1	1	2	2	2	2	2	2	2
104	62	5	0,63	1	71	H	1,7	67	23	2	1	2	2	2	2	2	2	2	2	2	2	2	2	2	2	2
105	31	5	0,63	9	79	M	1,54	67	28	2	1	2	2	1	2	2	2	1	2	2	2	2	1	2	2	2
106	59	4	0,63	2	74	H	1,62	86	32	2	2	2	2	2	2	2	2	2	2	2	2	2	2	2	2	2
107	86	5	0,63	2	62	M	1,47	77	35	1	1	2	1	2	2	2	2	1	2	2	2	2	2	2	2	2
108	43	5	0,64	1	66	H	1,62	62	23	2	1	2	2	1	2	2	2	1	2	2	2	2	2	2	2	2
109	82	6	0,64	1	64	H	1,62	93	35	1	1	2	2	2	1	2	2	2	2	2	2	2	2	2	2	2
110	63	6	0,65	3	70	M	1,52	57	24	2	1	1	2	2	2	2	2	1	1	2	2	2	2	2	1	2
111	59	7	0,65	3	66	M	1,55	75	31	1	1	1	2	1	2	1	2	1	2	1	2	2	1	2	1	2
112	38	5	0,67	4	73	H	1,66	91	33	2	1	2	2	2	2	2	2	1	2	2	2	2	2	2	2	2
113	70	6	0,67	7	68	H	1,7	103	35	2	2	2	2	2	2	1	2	1	2	2	2	2	2	1	1	2
114	95	6	0,68	3	48	H	1,73	64	21	2	2	2	2	2	2	2	2	2	2	2	2	2	2	2	2	2
115	75	5	0,68	2	65	H	1,65	86	31	2	2	1	2	2	2	2	2	1	2	2	2	2	2	2	1	2
116	40	5	0,69	2	76	H	1,72	75	25	2	1	1	2	1	2	2	2	2	2	2	2	2	2	2	2	2
117	117	7	0,71	1	80	M	1,55	54	22	2	1	2	2	2	2	2	2	1	2	2	2	2	1	2	1	2
118	105	7	0,72	6	78	M	1,57	87	35	1	1	1	2	2	2	2	2	1	2	2	2	2	2	2	1	2
119	42	7	0,74		70	M	1,47	34	15	2	1	2	2	2	2	2	2	2	2	2	2	2	2	2	2	2
120	98	5	0,76	1	73	M	1,44	70	33	2	1	2	2	2	2	2	2	2	1	2	2	2	1	2	2	2
121	83	7	0,77	1	44	H	1,75	82	26	1	2	2	2	1	2	1	2	2	1	2	2	2	1	1	1	2
122	106	4	0,77	1	83	M	1,6	67	26	1	2	2	2	2	2	2	2	1	2	1	2	2	2	2	2	2
123	70	6	0,79		65	H	1,55	71	29	2	2	2	2	2	2	2	2	2	2	2	2	2	2	2	2	2
124	118	5	0,8	9	50	M	1,59	70	27	2	1	1	2	2	1	2	1	1	2	2	2	2	2	2	2	2
125	39	6	0,82	1	82	H	1,61	80	30	1	1	1	2	1	2	2	2	2	2	2	2	2	1	2	1	1
126	78	6	0,86	4	44	M	1,64	67	25	2	2	2	2	2	2	2	2	2	2	2	2	2	2	2	2	2
127	20	5	0,87		73	H	1,82	69	20	2	2	2	2	2	2	2	2	1	2	2	2	2	2	2	2	2
128	78	5	0,87	1	33	M	1,56	55	22	2	2	2	2	2	1	2	2	2	2	2	2	2	2	2	2	2
129	82	5	0,87	2	76	H	1,66	82	29	2	1	1	2	2	1	1	2	2	2	1	2	2	2	1	2	2
130	46	5	0,88	1	74	H	1,69	79	27	2	1	2	1	1	2	2	2	1	2	1	2	2	2	2	2	2
131	42	6	0,92	1	87	M	1,48	54	25	1	1	1	1	2	2	2	2	2	2	1	2	2	2	2	2	2
132	60	5	0,92	2	41	H	1,7	76	26	2	1	2	2	2	2	2	2	2	2	2	2	2	2	2	2	2
133	106	6	0,93		72	M	1,48	60	27	2	1	1	2	2	2	2	2	2	2	2	2	2	1	2	1	2
134	61	5	0,94	3	82	M	1,55	56	23	2	2	2	2	2	2	2	2	2	2	2	2	2	1	2	2	2
135	121	7	0,94	1	73	H	1,69	86	30	2	1	1	2	1	2	2	2	1	2	2	2	1	2	2	1	2
136	112	6	0,95	1	42	H	1,73	83	27	2	2	1	2	2	2	2	2	2	2	2	2	2	1	1	1	2
137	25	5	0,95	1	76	M	1,63	80	30	2	2	1	2	2	2	2	2	1	2	2	2	2	1	2	1	2
138	72	5	0,96	7	74	M	1,67	65	23	2	2	2	2	2	2	1	2	2	2	2	1	2	1	2	1	2
139	93	6	0,96	1	51	M	1,56	59	24	2	2	2	2	2	2	2	2	2	2	2	2	2	2	2	2	2
140	43	5	0,98	4	60	M	1,43	78	38	2	1	1	2	2	1	2	1	1	1	1	2	2	2	2	1	2
141	88	6	0,99		69	H	1,69	67	23	2	1	1	1	2	2	2	1	2	2	2	2	2	2	2	1	2
142	40	5	0,99	3	75	M	1,73	81	27	2	1	1	1	2	2	2	2	2	2	1	2	1	2	2	1	2
143	78	6	1,01	3	68	H	1,67	60	21	2	2	2	2	2	2	2	2	2	2	2	2	2	1	2	2	2
144	60	5	1,02		75	M	1,55	52	21	2	2	2	2	1	2	2	2	1	1	2	2	1	2	2	2	2
145	100	6	1,02		74	H	1,8	97	29	1	2	1	2	1	2	2	2	1	2	2	2	1	2	2	2	1
146	45	5	1,02	4	74	H	1,72	93	31	2	2	2	2	2	1	1	2	1	2	2	2	2	1	2	2	2
147	36	5	1,03	9	81	M	1,6	66	26	2	2	1	2	2	2	2	2	1	2	2	2	2	1	2	1	2
148	20	4	1,03	2	82	M	1,65	80	29	1	1	1	2	1	2	2	2	1	2	2	2	2	1	2	1	2
149	32	4	1,03	4	58	M	1,53	96	41	2	2	2	2	1	2	2	2	2	2	2	2	2	2	2	2	2
150	59	5	1,04	9	71	H	1,8	93	28	2	1	2	2	1	2	2	2	1	2	2	2	2	2	2	2	2
151	19	5	1,06	1	57	M	1,53	105	44	1	2	2	2	1	2	2	2	1	2	2	2	2	2	2	1	2
152	61	6	1,07	2	56	H	1,64	79	29	1	1	2	2	1	2	2	2	2	2	2	2	2	2	2	2	2
153	36	5	1,07		69	H	1,71	91	31	2	2	2	2	2	2	2	2	2	2	2	2	2	2	2	2	2
154	40	6	1,08		75	H	1,71	73	24	2	1	2	2	2	2	2	2	2	2	2	2	2	1	2	2	2
155	78	6	1,08	6	50	M	1,71	94	32	2	1	2	2	2	2	2	2	2	2	2	2	2	2	2	2	2
156	114	6	1,1	1	76	M	1,55	55	22	2	1	1	2	2	2	2	2	1	2	2	2	2	1	2	1	2
157	102	5	1,11	1	63	M	1,54	68	28	2	2	2	2	2	2	2	2	2	2	2	2	2	2	2	2	2
158	61	6	1,14	1	52	H	1,78	94	29	2	2	2	2	2	2	2	2	2	2	2	2	2	2	2	2	2
159	76	6	1,15	3	60	H	1,84	88	25	2	2	1	2	2	2	2	2	2	2	2	2	2	2	2	2	2
160	62	6	1,16	6	78	H	1,82	86	25	2	2	2	2	2	2	2	2	1	1	2	2	2	2	2	2	2
161	46	5	1,17	3	49	H	1,65	74	27	2	1	2	2	2	2	2	2	2	2	2	2	2	1	2	1	2
162	62	6	1,18	8	49	M	1,57	67	27	2	2	2	2	2	2	2	2	2	2	2	2	2	2	2	2	2
163	100	5	1,18	1	54	M	1,71	80	27	2	1	2	2	2	2	2	2	2	2	2	2	2	2	2	2	2
164	31	5	1,19	2	53	H	1,62	60	22	1	2	2	2	2	2	2	2	2	2	2	2	2	2	2	2	2
165	93	6	1,24	4	51	H	1,65	64	23	2	2	2	2	2	2	2	2	2	2	2	2	2	2	2	2	2
166	43	5	1,26	1	74	H	1,7	67	23	2	2	1	2	2	2	1	2	2	2	2	2	2	2	1	1	2
167	62	6	1,27	6	62	H	1,59	87	34	2	1	2	2	1	2	2	2	2	2	2	2	2	2	2	2	2
168	81	5	1,28	1	67	M	1,5	56	25	1	2	1	1	2	2	2	2	2	2	2	2	2	1	2	1	1
169	48	4	1,34	1	74	M	1,49	74	33	1	1	1	1	2	2	1	2	1	2	2	2	1	2	2	2	2
170	82	6	1,37		75	M	1,48	72	32	2	2	2	2	2	2	2	2	1	2	2	2	2	2	2	2	2
171	41	5	1,39	0	66	M	1,64	81	30	1	2	2	2	1	2	2	2	1	2	2	2	2	2	2	2	2
172	21	6	1,39	1	72	M	1,49	73	33	2	1	2	2	1	2	2	1	1	2	2	2	2	1	2	2	2
173	35	5	1,4		58	H	1,58	62	24	2	1	1	2	2	2	2	2	1	2	2	2	2	2	2	1	2
174	55	6	1,42	2	75	M	1,58	52	20	1	2	2	2	2	2	2	2	1	1	2	2	2	2	2	2	2
175	18	4	1,51	3	48	M	1,75	160	52,2	2	2	1	2	1	1	2	2	1	2	2	2	1	2	1	2	2
176	95	6	1,56	5	62	H	1,76	92	30	1	1	1	2	2	1	2	2	1	2	2	2	2	1	2	1	1
177	38	4	1,57	5	63	H	1,57	70	28	2	1	1	2	2	2	1	2	2	2	2	2	2	1	1	1	2
178	63	5	1,67	3	69	H	1,65	57	21	2	1	2	1	2	2	2	2	2	2	2	2	2	2	2	2	2
179	42	5	1,69	1	82	H	1,63	75	28	2	1	2	2	2	2	2	2	2	2	2	2	2	1	2	2	2
180	18	6	1,74	1	73	M	1,47	71	32	2	1	1	2	2	2	2	2	2	2	2	2	2	2	2	2	2
181	63	6	2,04	2	61	H	1,63	74	28	2	1	2	2	2	2	2	1	1	1	1	2	2	2	2	2	2
182	98	6	2,23	2	68	H	1,57	77	31	2	1	1	2	1	2	1	2	1	2	2	2	2	2	2	1	2
183	60	5	2,53		76	H	1,62	82	31	2	1	2	2	2	2	2	2	1	2	2	2	2	2	2	2	2
184	60	5	2,91	7	62	M	1,56	72	29	2	1	2	2	2	2	2	2	1	1	2	2	2	1	2	2	2
185	80	4	2,93	0	62	M	1,5	72	32	2	1	2	2	2	2	2	2	2	2	2	2	2	2	2	2	2
186	111	5	3,15	3	65	H	1,77	75	23	2	2	2	1	2	1	2	2	2	2	2	2	2	1	2	2	2
187	103	6	3,64	4	54	H	1,7	70	24	2	1	2	2	2	1	2	2	2	2	2	2	2	1	2	2	2
188	58	5	6,54	2	53	H	1,69	88	30	2	1	2	2	1	2	2	2	1	2	2	2	2	2	2	2	2

Dataset. Relationship between Urinary Level of Phytate and Valvular Calcification in an Elderly Population: a Cross-Sectional Study

Subject	Ezetimibe_1yes_2not	IECAS_1yes_2not	ARAII_1yes_2not	Diur.Prox_1yes_2not	Diur.Dist_1yes_2not	BetaBl_1yes_2not	ADOs_1yes_2not	Insuline_1yes_2not	ACa_1yes_2not	Calcitonine_1yes_2not	Calcium_1yes_2not	TSH_1yes_2not	Smoking_1yes_2no_3ex	Excercise_1not_2smt_3usual	Alcohol_1yes_2smt_3not	Smoker_1yes_2not or ex	Excercise__1yes_2not	Albumin	ALP	ALT	AST	Basophils
1	2	1	2	2	1	2	1	2	2	1	2	2	2	1	3	2	2	41	151	16	23	0,5
2	2	2	1	2	1	2	2	1	1	2	1	2	3	2	3	1	1	38	102	27	21	0,5
3	2	2	2	2	2	1	2	2	2	2	2	2	1	1	2	1	2	40	91	15	24	0,7
4	2	2	2	2	2	2	2	2	2	2	2	2	2	3	1	2	2	46	90	19	16	0,4
5	2	2	2	2	2	2	2	2	2	2	2	2	2	1	3	2	2	44	101	28	17	0,2
6	2	2	2	2	1	2	2	2	2	2	2	2	2	3	2	2	2	41	48	41	30	0,4
7	2	1	2	2	2	2	2	2	2	2	2	2	3	2	1	1	1	41	44	139	60	0,1
8	2	2	2	2	2	2	2	2	2	2	2	2	2	1	3	2	2	28	57	9	11	0,4
9	2	1	2	2	1	1	2	2	1	2	2	2	1	1	1	1	2	41	75	9	12	0,5
10	2	2	2	2	2	2	2	2	1	2	2	2	3	3	1	1	2	37	90	51	40	0,2
11	2	2	1	2	1	1	2	2	2	2	2	2	2	3	1	2	2	46	47	16	32	0,8
12	2	2	2	2	2	2	2	2	1	2	2	2	2	2	2	2	1	41	87	25	18	3,0
13	2	2	2	2	2	2	2	2	2	2	2	2	1	3	1	1	2	44	65	18	22	0,4
14	2	2	2	2	2	2	2	2	2	2	2	2	2	2	1	2	1	42	79	39	23	0,8
15	2	1	2	2	2	2	2	1	2	2	2	2	2	3	3	2	2	46	63	22	18	0,4
16	2	2	2	2	2	2	2	2	2	2	2	2	2	1	3	2	2	39	69	12	17	0,3
17	2	2	2	2	1	2	2	2	2	2	2	2	1	3	2	1	2	34	66	74	28	0,1
18	2	2	2	2	2	2	2	2	2	2	2	2	2	1	3	2	2	39	134	45	31	0,6
19	2	1	2	2	1	1	2	1	1	2	2	2	3	3	2	1	2	35	56	14	21	0,2
20	2	1	2	2	1	2	2	2	2	2	1	2	2	2	2	2	1	41	51	16	16	0,3
21	2	2	2	2	2	2	1	1	1	2	2	2	2	1	1	2	2	43	136	23	22	0,4
22	2	1	2	2	2	2	2	2	1	2	2	2	2	3	3	2	2	44	81	28	19	1,4
23	2	1	2	2	2	2	2	1	1	2	2	2	2	3	1	2	2	46	56	27	18	0,6
24	2	1	2	2	2	1	2	2	2	2	2	2	2	2	3	2	1	44	89	8	14	0,5
25	2	2	2	2	2	2	2	2	2	1	1	2	2	3	1	2	2	47	80	15	26	0,5
26	2	2	1	2	1	1	1	2	2	1	2	2	2	1	3	2	2	42	52	18	16	0,6
27	2	2	2	2	2	1	2	2	2	2	2	2	3	2	3	1	1	45	95	42	35	0,3
28	2	2	1	1	2	2	2	2	1	2	2	2	2	3	3	2	2	45	87	16	19	0,3
29	2	2	2	2	2	2	2	2	2	2	2	2	3	3	3	1	2	38	114	18	18	3,0
30	2	2	2	2	2	2	2	2	2	2	2	2	2	2	3	2	1	44	103	14	15	0,4
31	2	2	2	1	2	1	1	2	1	2	2	2	2	2	3	2	1	27	112	25	25	0,2
32	2	2	2	2	2	1	2	2	1	2	2	2	2	2	3	2	1	46	66	18	17	0,7
33	2	2	2	1	2	2	1	2	1	2	2	2	2	2	3	2	1	45	104	22	21	0,4
34	2	2	2	2	2	2	1	2	2	2	2	2	1	1	3	1	2	41	38	17	15	0,2
35	2	1	2	1	2	2	1	1	1	2	2	2	2	1	3	2	2	38	87	31	19	0,4
36	2	2	1	2	2	2	2	2	2	2	2	2	2	2	3	2	1	46	69	17	20	0,5
37	2	1	2	2	2	1	2	2	2	2	2	2	2	3	2	2	2	43	70	16	21	0,7
38	2	1	2	2	1	1	2	2	2	2	2	2	2	3	3	2	2	44	47	31	28	0,6
39	2	2	2	2	2	2	2	1	2	2	2	2	3	3	3	1	2	37	43	9	11	0,2
40	2	1	2	2	2	1	1	1	1	2	2	2	2	1	3	2	2	46	54	23	19	0,9
41	2	2	1	2	2	1	2	2	2	2	2	2	2	3	3	2	2	42	93	50	38	0,4
42	2	2	2	2	2	2	2	2	2	2	2	2	2	2	3	2	1	45	64	13	15	0,6
43	2	1	2	1	2	2	2	1	2	2	2	2	2	3	3	2	2	41	59	62	41	0,6
44	2	1	2	2	1	1	1	2	2	2	2	2	1	1	1	1	2	40	81	11	12	0,3
45	2	2	2	2	2	2	2	2	2	2	2	2				2	2	41	62	12	19	0,4
46	2	2	2	2	2	2	2	2	2	2	2	2	1	1	3	1	2	41	91	10	13	0,5
47	2	2	1	2	1	2	2	2	2	2	2	2	2	2	3	2	1	42	96	15	18	0,5
48	2	2	1	2	1	1	1	2	2	2	2	2	1	1	3	1	2	17	123	28	17	0,1
49	2	2	2	2	2	2	2	2	2	2	2	2	2	3	1	2	2	45	77	12	18	0,2
50	2	2	2	2	2	1	2	2	2	2	1	2	3	2	2	1	1	38	49	16		0,6
51	2	1	2	2	1	1	1	1	1	2	2	2	3	3	3	1	2	34	114	16	17	0,7
52	2	2	2	2	2	2	2	2	2	2	2	2				2	2	42	113	131	58	0,4
53	2	2	2	2	2	2	2	2	2	2	2	2				2	2	39	71	12	16	0,5
54	2	2	2	2	2	2	2	2	1	2	2	2	2	3	2	2	2	42	117	16	19	0,6
55	2	1	2	2	1	2	1	1	2	2	2	2	2	1	3	2	2	45	105	41	61	0,1
56	2	1	2	2	2	2	2	2	2	2	2	2	1	2	2	1	1	43	80	18	18	0,3
57	2	2	2	2	2	2	2	2	2	2	2	2	2	3	1	2	2	44	58	22	18	0,5
58	2	2	2	2	2	2	2	2	2	2	2	2	1	1	1	1	2	47	62	20	17	0,7
59	2	2	1	1	2	2	1	2	2	2	2	2	2	3	1	2	2	43	56	12	19	0,4
60	2	2	1	2	1	2	1	2	2	2	2	2	2	3	3	2	2	32	89	54	52	0,6
61	2	1	2	2	2	2	2	2	2	2	2	2	2	2	3	2	1	48	94	23	23	0,5
62	2	2	2	2	2	2	2	2	2	2	2	2	1	3	1	1	2	44	84	49	40	0,4
63	2	2	2	2	2	2	2	2	2	2	2	2	2	1	3	2	2	43	113	25	26	0,7
64	2	2	1	2	1	2	1	2	2	2	2	2	2	1	3	2	2	42	83	13	14	0,7
65	2	2	2	2	2	1	2	2	2	2	2	2	2	3	1	2	2	48	62	21	17	0,4
66	2	1	2	2	2	2	2	2	2	2	2	2	3	2	3	1	1	40	64	14	13	0,8
67	2	2	1	2	2	2	2	2	2	2	2	2	2	1	3	2	2	40	57	69	61	0,5
68	2	2	2	2	2	2	2	2	2	2	2	2	2	2	3	2	1	30	119	13	13	0,2
69	2	2	2	1	2	1	2	2	2	2	2	2	3	3	3	1	2	45	49	31	27	0,9
70	2	2	2	2	2	2	2	2	2	2	2	2	2	3	1	2	2	46	79	14	16	0,3
71	2	2	2	2	2	1	2	2	2	2	2	2	2	3	3	2	2	42	80	22	22	0,7
72	2	1	2	2	2	2	1	2	1	2	2	2	2	3	1	2	2	41	61	14	15	0,6
73	2	1	2	2	2	2	1	1	2	2	2	2	2	1	3	2	2	44	79	16	16	0,4
74	2	1	2	2	1	1	2	2	1	2	2	2	2	1	1	2	2	44	77	25	24	0,9
75	2	2	2	1	2	2	2	2	2	2	2	2	3	1	1	1	2	42	84	16	20	0,4
76	2	2	2	2	2	2	2	2	2	2	2	2	3	1	2	1	2	48	58	36	35	0,4
77	2	2	2	2	1	2	2	2	2	2	2	2	2	3	1	2	2	45	56	18	18	0,8
78	2	2	1	2	2	2	1	2	2	1	2	2	2	3	3	2	2	43	49	21	17	0,2
79	2	2	1	2	2	2	1	1	1	2	2	2	2	1	3	2	2	42	69	20	17	0,3
80	2	1	2	2	2	2	2	2	1	2	2	2	2	2	2	2	1					0,3
81	2	2	1	2	1	2	2	2	2	2	2	2	1	1	1	1	2	46	118	26	29	0,3
82	2	1	2	2	2	2	2	2	2	2	2	2	2	3	3	2	2	41	56	13	17	0,4
83	2	2	2	2	2	2	2	2	2	2	2	2	2	3	3	2	2	44	62	21	23	0,4
84	2	2	2	2	1	2	2	2	1	2	2	2	2	2	3	2	1	40	62	17	17	0,3
85	2	2	2	2	2	1	2	2	2	2	2	2	1	2	3	1	1	42	49	12	16	0,9
86	2	1	2	2	2	2	2	2	2	2	2	2	2	2	3	2	1	45	83	9	20	0,6
87	2	2	1	2	2	1	1	1	2	2	2	2	2	1	3	2	2	43	89	19	19	0,4
88	2	2	2	2	2	2	2	2	2	2	2	2	2	1	2	2	2	46	57	23	22	0,5
89	2	2	1	2	2	2	2	2	1	2	2	2	3	1	3	1	2	48	95	14	13	0,4
90	2	2	2	2	2	2	2	2	1	2	2	2	2	2	3	2	1	38	72	37	17	0,3
91	2	2	2	2	2	2	2	2	2	2	2	2	2	1	1	2	2	47	50	7	14	0,3
92	2	2	2	2	2	1	2	2	2	2	2	2	2	2	2	2	1	42	43	17	16	0,5
93	2	2	2	2	2	2	2	2	2	2	2	2	2	1	3	2	2	45	96	16	17	0,4
94	2	2	2	2	2	1	2	2	2	2	2	2	2	3	3	2	2	40	53	21	25	0,1
95	2	2	2	2	2	2	2	2	2	2	2	2	2	2	3	2	1	41	98	14	20	0,7

Dataset. Relationship between Urinary Level of Phytate and Valvular Calcification in an Elderly Population: a Cross-Sectional Study

Subject	Ezetimibe_1yes_2not	IECAS_1yes_2not	ARAII_1yes_2not	Diur.Prox_1yes_2not	Diur.Dist_1yes_2not	BetaBl_1yes_2not	ADOs_1yes_2not	Insuline_1yes_2not	ACa_1yes_2not	Calcitonine_1yes_2not	Calcium_1yes_2not	TSH_1yes_2not	Smoking_1yes_2no_3ex	Excercise_1not_2smt_3usual	Alcohol_1yes_2smt_3not	Smoker_1yes_2not or ex	Excercise__1yes_2not	Albumin	ALP	ALT	AST	Basophils
96	2	1	1	2	2	2	1	2	1	2	2	2	2	1	3	2	2	40	85	32	28	1,1
97	2	2	1	2	1	2	2	2	1	2	2	2	2	3	3	2	2	44	83	15	20	0,9
98	2	1	2	2	1	2	2	1	2	2	2	2	1	3	1	1	2	39	76	20	27	0,3
99	2	1	2	2	1	1	2	2	2	2	2	2	3	2	3	1	1	31	46	49	23	0,2
100	2	1	2	2	2	1	2	2	2	2	2	2	2	3	3	2	2	44	47	13	12	0,0
101	2	2	2	2	2	2	2	2	2	2	2	2	2	2	3	2	1	45	80	12	14	0,3
102	2	1	2	2	2	2	2	2	2	2	2	2	3	2	1	1	1	45	63	17	21	0,5
103	2	2	2	1	2	1	2	2	2	2	1	2	2	2	3	2	1	45	60	21	19	0,7
104	2	2	2	2	2	2	2	2	2	2	2	2	3	2	3	1	1	31	114	19	15	0,8
105	2	2	2	1	2	2	2	2	2	2	2	2	2	1	3	2	2	45	116	12	17	0,3
106	2	2	2	2	2	2	2	2	2	2	2	2				2	2	50	37	36	28	0,5
107	2	2	1	2	1	2	2	1	2	2	2	2	2	1	2	2	2	38	132	13	9	0,7
108	2	2	2	2	1	2	2	2	2	2	2	2	1	3	3	1	2	44	74	15	22	1,5
109	2	1	2	2	2	1	1	2	2	2	2	2	2	1	1	2	2	41	63	24	24	0,5
110	2	2	2	2	2	2	2	2	1	2	2	2	2	2	1	2	1	44	60	19	18	0,4
111	2	1	2	1	1	1	1	1	2	2	2	2	2	1	3	2	2	41	95	16	15	0,4
112	2	1	2	2	2	1	2	2	2	2	2	2	2	3	3	2	2	45	105	23	27	0,4
113	2	2	2	2	2	1	2	2	2	2	2	2	2	3	3	2	2	43	86	15	18	0,3
114	2	2	2	2	2	2	2	2	2	2	2	2	2	3	3	2	2	47	46	25	22	0,4
115	2	2	2	2	1	1	2	2	2	2	2	2	3	2	2	1	1	43	61	17	20	0,5
116	2	1	2	2	2	2	2	2	2	2	2	2	2	3	2	2	2	41	104	15	15	0,3
117	2	1	2	2	2	1	2	2	2	2	2	2	1	1	3	1	2	34	134	17	20	0,4
118	2	1	2	2	1	2	2	1	1	2	2	2	2	2	3	2	1	45	62	14	17	0,6
119	2	2	2	2	2	2	2	2	2	2	2	2	2	2	2	2	1	46	65	18	28	0,8
120	2	2	1	1	2	1	2	2	2	2	1	2	2	1	3	2	2	43	104	13	16	0,8
121	2	2	2	2	2	2	2	1	2	2	2	2	3	3	2	1	2	41	61	21	17	0,6
122	2	2	2	2	2	1	2	2	2	2	2	2	2	2	3	2	1	45	74	15	18	0,5
123	2	2	2	2	2	2	2	2	2	2	2	2	2	2	1	2	1	45	62	20	22	1,2
124	2	1	1	2	1	2	2	2	2	2	2	2	2	1	3	2	2	45	87	13	14	0,5
125	2	1	2	2	2	2	2	2	1	2	2	2	2	3	3	2	2	46	29	29	26	0,8
126	2	2	2	2	2	2	2	2	2	2	2	2	2	1	3	2	2	43	70	6	12	0,5
127	2	2	2	2	2	2	2	2	2	2	2	2	1	3	1	1	2	40	84	13	19	0,2
128	2	1	2	2	2	1	2	2	2	2	2	2	1	1	3	1	2	40	33	10	10	0,7
129	1	2	2	2	2	1	2	2	1	2	2	2	3	3	2	1	2	43	79	15	17	0,5
130	2	1	2	2	2	2	2	2	2	2	2	2	3	3	3	1	2	41	55	11	14	0,5
131	2	2	2	2	2	2	2	2	2	2	2	2	2	1	3	2	2	35	177	24	16	0,2
132	2	1	2	2	2	1	2	2	2	2	2	2	2	3	1	2	2	45	68	19	20	0,5
133	2	2	2	2	2	2	2	2	2	2	2	2	2	2	1	2	1	48	65	7	19	0,2
134	2	1	2	2	1	1	2	2	2	2	2	2	2	3	3	2	2	38	69	56	47	0,4
135	2	2	2	2	1	2	2	2	2	2	2	2	3	3	2	1	2	37	81	32	18	0,3
136	2	2	2	2	2	1	2	2	2	2	2	2	1	2	2	1	1	45	80	175	105	0,7
137	2	2	2	2	2	2	2	2	2	2	2	2	3	2	2	1	1	39	73	32	15	0,5
138	2	1	2	2	2	1	2	2	2	2	2	2	2	2	3	2	1	36	67	81	93	0,4
139	2	2	2	2	2	2	2	2	2	2	2	2	2	3	2	2	2	45	47	15	14	0,6
140	2	2	1	2	1	1	2	2	2	2	1	2	2	1	3	2	2	42	85	23	21	0,5
141	2	2	1	2	2	2	2	2	1	2	2	2	1	1	2	1	2	44	138	23	16	0,5
142	2	1	2	2	1	1	2	2	2	2	2	2	2	2	3	2	1	37	78	17	16	0,9
143	2	1	2	2	1	2	2	2	2	2	2	2	3	1	3	1	2	37	56	59	23	0,4
144	2	2	2	2	2	2	2	2	2	2	2	2	1	3	3	1	2	42	206	12	18	0,4
145	2	2	2	2	2	2	1	2	2	2	2	2	3	3	3	1	2	47	50	23	14	0,3
146	2	1	2	2	2	1	2	2	2	2	2	2	2	2	3	2	1	43	52	15	17	1,8
147	2	2	2	2	2	2	2	2	2	2	2	2	2	1	2	2	2	39	85	15	21	0,8
148	2	1	2	2	1	2	1	2	2	2	2	2	2	1	3	2	2	44	62	18	18	0,6
149	2	2	2	2	2	2	2	2	2	2	2	2	2	1	3	2	2	43	67	21	20	0,5
150	2	2	2	2	1	2	2	2	1	2	2	2	3	2	1	1	1	45	46	23	22	0,4
151	2	2	2	2	2	2	1	1	2	2	2	2	2	1	3	2	2	41	55	16	13	0,9
152	2	2	1	2	2	1	2	2	2	2	2	2	2	3	2	2	2	47	38	12	14	0,5
153	2	2	2	2	2	2	2	2	2	2	2	2	2	1	1	2	2	47	61	18		0,2
154	2	2	2	2	2	2	2	2	2	2	2	2	2	1	3	2	2	41	64	17	16	0,6
155	2	2	2	1	2	2	2	2	2	2	2	2	2	2	3	2	1	42	52	16	12	1,2
156	2	1	2	2	1	1	2	2	2	2	2	2	2	2	3	2	1	35	89	14	14	0,5
157	2	2	2	2	2	2	2	2	2	1	2	2	2	3	2	2	2	43	55	21	18	0,4
158	2	2	2	2	2	2	2	2	2	2	2	2	1	2	2	1	1	44	66	27	19	0,6
159	2	2	2	2	2	2	2	2	2	2	2	2	2	1	2	2	2	44	68	40	26	0,2
160	2	2	2	2	1	2	2	2	2	2	2	2	2	3	2	2	2	41	60	29	24	0,7
161	2	2	2	2	2	1	2	2	2	2	2	2	2	3	2	2	2	46	42	19	19	0,8
162	2	2	2	2	2	2	2	2	2	2	2	2				2	2	42	60	16	16	0,4
163	2	2	2	2	2	2	2	2	2	2	2	2	1	3	1	1	2	44	79	29	22	0,6
164	2	2	2	2	1	2	2	2	2	2	2	2	1	1	3	1	2	40	125	23	28	0,8
165	2	2	2	2	2	2	2	2	2	2	2	2	1	1	3	1	2	48	96	20	16	0,4
166	2	1	2	2	2	2	2	2	2	2	2	2	2	3	3	2	2	43	75	16	19	0,5
167	2	2	1	2	2	2	2	2	1	2	2	2	2	2	3	2	1	45	69	21	15	0,5
168	2	2	2	2	2	1	1	1	2	2	2	2	2	1	3	2	2	45	73	18	19	0,7
169	2	2	2	2	1	2	2	1	2	2	2	2	2	1	3	2	2	33	109	18	20	0,2
170	2	2	2	2	2	2	2	2	2	2	2	2	2	1	1	2	2	46	73	9	14	0,6
171	2	2	2	2	2	1	2	2	2	2	2	2	2	3	3	2	2	44	97	28	22	1,1
172	2	2	1	2	2	2	2	2	1	2	2	2	2	2	3	2	1	43	91	20	15	0,3
173	2	2	2	2	2	2	2	2	2	2	2	2	2	2	2	2	1	44	91	20	18	0,3
174	2	2	2	2	2	2	2	2	2	2	2	2	2	2	3	2	1	43	70	21	22	0,5
175	2	2	2	2	2	2	2	2	2	2	2	2	2	3	3	2	2	40	63	20	18	0,2
176	2	1	2	2	1	2	1	2	2	2	2	2	2	3	3	2	2	45	54	48	20	0,5
177	2	1	2	1	2	1	2	2	2	2	2	2	2	3	2	2	2	45	67	40	30	1,1
178	2	2	1	2	1	2	2	2	2	2	2	2	2	3	3	2	2	39	82	27	32	0,5
179	2	2	1	2	1	2	2	2	2	2	2	2	2	3	2	2	2					0,5
180	2	1	2	2	1	2	2	2	2	2	2	2	2	2	3	2	1	44	86	12	12	0,5
181	2	1	2	2	1	2	2	2	2	2	2	2	2	1	1	2	2	36	66	50		0,2
182	2	2	2	2	2	2	2	2	2	2	2	2	2	3	3	2	2	38	83	22		0,2
183	2	1	2	2	2	2	2	2	2	2	2	2	1	1	1	1	2	43	36	11	16	0,3
184	2	2	1	1	2	1	2	2	2	2	1	2	2	2	3	2	1	41	163	24	17	0,2
185	2	2	2	2	2	1	2	2	1	2	2	2	2	2	3	2	1	45	63	16	13	0,5
186	2	1	2	2	2	2	2	2	2	2	2	2	1	1	3	1	2	39	75	10	10	0,7
187	2	1	2	2	2	2	2	2	2	2	2	2	2	3	1	2	2	45	74	22	15	0,8
188	2	1	2	2	2	2	2	2	2	2	2	2	2	2	2	2	1	45	63	13	18	0,5

Dataset. Relationship between Urinary Level of Phytate and Valvular Calcification in an Elderly Population: a Cross-Sectional Study

Subject	BilirrubinT	Calcium	Chloride	Cholesterol Total	Creatinine	Eosinophiles	Fibrinogen	Phosphorous	GGT	Glycemia	Haemoglobine	Homocysteine	HDL	Hematíes (10^6)	Haematocrit	LDL-C	Leukocytes	Lymphocytes
1	0	10	95	150	1	3	503	3	277	130	11	16	64	3	34	67	5	26
2	0	9	98	120	4	1	510	6	130	91	9	19	42	3	28	53	7	22
3	1	9	99	155	0	1	305	4	30	95	13	5	61	4	37	79	8	15
4	0	9	100	206	0	1	365	3	24	112	14	8	58	4	43	130	7	34
5	0	10	98	269	0	1	450	3	28	120	14	6	59	4	42	179	8	38
6	1	9	102	162	0	0	433	3	23	131	15	8	65	4	43	83	6	17
7	1	9	94	169	0	3	362	4	101	143	15	9	45	5	47	101	7	21
8	0	8	92	118	1	3	660	3	13	149	9	13	50	3	29	34	9	16
9	0	9	107	200	2	8	385	4	17	93	10	9	40	3	33	137	6	25
10	0	8	99	163	0	3	532	4	45	94	11	4	61	3	34	88	3	21
11	0	9	95	200	0	5	356	3	18	134	13	9	68	4	39	99	5	32
12	0	9	110	125	2	2	434	3	22	94	10	10	23	3	30	74	7	16
13	0	8	99	166	0	2	263	4	21	110	13	10	71	4	41	82	6	33
14	0	9	101	176	1	2	313	4	30	101	14	12	42	5	40	106	6	24
15	0	8	99	199	0	2	372	4	15	155	12	4	69	4	35	102	6	33
16	0	9	109	213	2	4	522	1	10	114	11	6	46	3	34	119	5	24
17	0	8	98	134	1	0	440	3	65	76	11	17	58	4	37	62	9	13
18	0	11	95	219	1	1	461	3	109	125	11	28	44	4	35	119	6	18
19	0	9	103	201	1	1	512	3	15	109	10	11	41	3	32	137	3	36
20	0	10	101	165	1	1	365	4	15	109	13	30	62	4	40	80	7	16
21	0	9	99	156	0	4	494	3	157	182	14	8	48	4	42	81	8	29
22	0	9	105	242	0	1	366	4	33	122	12	5	53	4	36	156	6	35
23	0	10	96	144	1	3	382	3	14	156	15	7	43	5	45	80	6	40
24	0	9	99	163	4	2	390	5	22	100	12	35	52	4	39	79	14	56
25	0	9	106	241	0	2	266	3	20	90	13	8	44	4	40	152	5	33
26	0	9	104	250	1	3	410	4	12	144	11	6	48	4	33	169	5	34
27	0	10	97	128	1	3	407	3	56	103	14	12	31	5	45	71	8	28
28	0	10	100	167	1	2	410	3	17	115	14	8	42	4	40	102	8	20
29	1	9	104	134	1	0	327	3	17	108	8	3	23	2	26	88	134	84
30	0	9	104	284	0	7	345	3	16	89	14	7	96	4	42	173	7	42
31	0	8	104	158	2	0	295	3	45	125	11	13	22	3	33	95	13	9
32	1	9	104	134	1	3	315	4	28	121	14	7	39	4	41	52	7	30
33	0	10	99	157	1	1	351	2	16	146	13	9	62	4	40	67	7	28
34	0	8	104	141	1	3	410	3	12	85	11	15	32	3	33	89	5	28
35	0	9	95	258	1	3	428	4	68	171	10	6	66	4	33	159	10	21
36	0	9	100	211	1	2	363	3	18	102	13	11	77	4	41	121	7	36
37	0	9	103	185	1	6	356	3	31	99	10	6	52	4	31	105	7	21
38	0	8	100	172	0	4	312	2	19	94	14	7	61	5	41	86	12	34
39	1	8	102	146	1	1	602	2	19	103	8	7	33	2	24	89	2	43
40	0	9	99	180	2	3	388	4	20	118	14	14	37	4	43	58	6	27
41	0	9	97	244	1	2	314	3	65	288	12	18	54	4	38	155	13	20
42	0	10	100	162	0	3	372	4	17	90	13	7	63	4	38	85	8	26
43	0	9	98	151	0	1	312	4	35	168	13	3	96	4	38	34	4	30
44	0	9	101	239	0	3	589	3	34	253	14	11	37	5	44	167	8	32
45	0	9	101	125	1	11	513	3	20	102	12	8	44	4	37	34	7	37
46	0	9	103	195	0	2	413	3	12	95	14	11	49	4	42	121	7	34
47	1	9	108	189	0	3	400	2	10	101	14	8	72	4	41	102	4	36
48	0	7	101	151	1	1	998	3	59	160	6	8	40	2	20	89	17	9
49	0	9	100	185	0	2	317	3	12	96	13	4	83	4	40	90	5	27
50		9	101	239	0	1	239	4	32	86	13	11	60	3	39	123	7	27
51	0	9	98	149	1	3	750	5	71	105	11	12	32	4	35	71	20	31
52	0	9	102	213	0	2	380	3	140	104	13	5	64	4	40	127	4	32
53	0	9	101	199	0	5	429	3	13	87	12	6	77	5	40	97	6	30
54	0	8	101	209	0	4	439	2	18	113	15	3	48	5	46	143	6	23
55	1	9	94	283	0	0	538	3	58	168	12	13	61	5	39	179	6	14
56	0	9	109	176	0	1	299	4	16	101	14	11	75	4	43	88	9	28
57	0	9	104	175	1	3	340	3	23	100	14	5	38	5	42	109	5	37
58	0	10	103	119	0	2	499	4	37	110	15	7	40	5	45	36	9	25
59	0	9	103	188	1	3	407	3	19	151	11	15	55	3	33	101	6	39
60	1	8	109	153	0	1	206	3	288	103	11	10	69	3	35	67	7	24
61	0	10	100	199	1	4	446	4	82	102	12	23	68	4	37	107	6	41
62	0	10	104	187	0	1	275	2	166	104	12		69	4	39	97	9	27
63	0	9	102	149	0	3	172	3	21	105	13	11	72	4	40	66	5	30
64	0	9	95	173	0	4	583	4	15	187	11	10	49	4	35	98	8	29
65	0	9	104	208	0	2	261	3	39	97	14	6	87	4	42	107	4	34
66	0	9	104	204	0	2	351	3	25	97	14	4	71	4	41	115	5	37
67	0	8	103	185	1	3	287	3	32	90	10	21	57	3	34	98	2	35
68	0	8	106	211	0	2	480	3	58	88	9	31	39	3	30	150	9	14
69	0	9	103	225	0	4	320	4	19	100	15	5	69	5	45	131	5	46
70	0	9	104	225	0	2		3	12	107	13	5	66	4	40	142	5	25
71	1	9	104	204	1	2	371	3	73	113	15	7	57	5	47	128	6	31
72	0	9	102	201	1	6	538	3	16	131	11	9	47	4	37	134	7	32
73	0	9	101	160	1	2	368	3	16	248	12	7	43	4	38	82	9	36
74	0	9	102	198	0	7	636	3	38	117	13	11	58	5	41	67	7	23
75	0	9	105	229	1	5	358	2	19	104	13	7	54	4	39	153	6	27
76	0	9	107	173	0	1	373	3	36	92	12	50	83	3	40	79	7	38
77	0	9	105	224	1	7	313	3	42	106	14	8	63	5	44	148	6	28
78	0	9	101	199	0	3	248	4	29	150	12	6	51	4	38	106	8	34
79	0	9	98	225	0	4	554	3	19	170	12	7	52	4	37	147	9	35
80			111			2	380				12			4	36	0	5	26
81	0	9	97	237	0	4	372	3	47	96	16	10	48	5	48	136	10	23
82	0	9	102	199	1	2	571	3	13	111	14	13	49	4	42	132	7	29
83	1	9	101	185	0	0	458	3	15	106	16	12	40	5	45	120	11	15
84	0	9	96	202	1	1	426	3	20	94	13	21	62	4	38	111	12	22
85	0	9	108	156	1	1	275	3	8	97	13	13	33	4	42	103	7	21
86	0	9	102	224	1	1	349	3	27	88	13	14	69	4	40	140	9	27
87	0	9	102	162	1	6	436	4	16	87	10	14	37	3	31	102	6	34
88	1	9	102	250	0	4	330	2	20	101	13	6	49	4	40	185	5	29
89	0	9	102	160	0	2	508	3	31	110	13	6	67	4	42	69	7	34
90	0	9	93	108	1	2	855	3	27	103	12	6	39	4	36	47	14	9
91	0	9	101	177	0	1	242	3	10	83	13	8	71	4	39	98	7	30
92	0	9	98	220	0	1	270	3	33	164	14	4	39	5	42	147	5	25
93	0	9	99	236	0	5	304	4	9	87	12	6	48	4	36	156	6	23
94	1	9	101	181	0	0	508	3	32	98	12	6	58	4	36	111	7	12
95	0	8	102	173	0	3	481	4	12	89	12	5	75	4	35	83	7	28

Dataset. Relationship between Urinary Level of Phytate and Valvular Calcification in an Elderly Population: a Cross-Sectional Study

Subject	BilirrubinT	Calcium	Chloride	Cholesterol Total	Creatinine	Eosinophiles	Fibrinogen	Phosphorous	GGT	Glycemia	Haemoglobine	Homocysteine	HDL	Hematíes (10^6)	Haematocrit	LDL-C	Leukocytes	Lymphocytes
96	0	9	100	195	0	3	340	3	48	110	14	10	57	4	42	114	6	30
97	0	9	100	192	1	1	372	3	24	124	14		39	4	41	133	5	18
98	1	8	94	141	1	1	352	5	86	107	14	10	31	4	39	95	10	24
99	0	8	109	142	1	0	440	3	24	104	9	18	44	3	27	74	5	7
100	0	9	104	163	0	0	257	2	14	102	13	8	41	4	40	88	4	29
101	0	9	98	268	1	2	447	3	27	133	15	10	59	5	44	193	8	25
102	0	9	107	225	1	3	286	3	30	110	45	14	48	5	48	155	6	26
103	0	9	97	177	0	5	364	3	17	111	12	8	53	4	37	100	6	32
104	0	9	98	175	1	5	475	3	158	106	9	11	29	3	28	81	6	30
105	0	9	98	214	0	0	320	3	20	147	12	9	52	4	37	142	9	27
106	1	10	103	241	1	1	390	3	71	154	13	10	52	4	41	99	6	38
107	0	10	104	166	1	1	407	3	55	176	12	6	39	4	37	85	13	32
108	0	9	98	221	0	3	423	2	27	109	14	7	52	4	41	145	8	28
109	1	9	95	203	1	2	367	3	48	103	16	11	52	5	46	117	6	35
110	0	9	106	164	0	2	363	4	42	104	12	4	64	4	36	90	5	37
111	0	9	104	126	1	0	447	3	14	43	13	13	55	5	42	56	7	16
112	1	9	103	173	1	2	348	3	20	111	16	7	50	5	49	101	6	30
113	0	8	105	151	0	2	484	3	13	94	13	7	46	4	40	86	7	28
114	1	9	100	206	0	4	243	4	14	90	14	7	53	4	43	139	4	29
115	0	9	105	207	1	2	319	4	17	113	13	8	37	4	40	101	7	37
116	0	8	102	188	1	3	333	2	19	94	15	105	48	5	46	108	4	34
117	0	9	99	144	1	3	442	4	64	91	10	17	52	3	31	74	9	15
118	0	9	100	183	0	1	433	3	32	142	10	4	58	4	33	105	8	45
119	0	9	106	218	0	2	279	4	26	93	12	9	115	4	40	88	4	28
120	0	9	103	167	0	2	329	4	11	94	12	6	48	4	38	95	5	33
121	0	9	96	168	0	5		4	29	191	12	4	35	4	36	113	11	33
122	0	9	100	169	0	1	383	4	31	156	12	14	48	4	37	95	7	24
123	1	9	104	172	0	6	430	3	17	84	15	7	73	5	48	88	4	28
124	0	9	99	253	0	2	375	4	34	92	11	9	39	4	32	154	4	32
125	0	9	105	138	1	2	308	2	11	115	14	16	42	5	42	82	7	23
126	0	9	106	166	0	1	333	3	8	94	11	19	44	4	35	95	8	27
127	0	9	100	198	0	1	570	4	29	90	15	17	58	4	47	125	4	28
128	0	10	109	188	1	1	299	3	9	79	14	9	83	4	43	83	8	40
129	0	9	105	208	1	2	402	3	21	111	14	12	49	4	43	140	6	24
130	0	8	103	170	0	4	346	3	21	82	13	5	45	4	40	108	7	33
131	0	9	95	119	3	3	865	7	14	201	9	27	44	3	28	35	12	21
132	0	9	101	114	0	0	310	3	16	98	14	5	59	5	42	45	12	13
133	0	10	104	271	0	0		3	13	78	14	7	106	4	45	136	5	45
134	0	9	103	186	0	2	455	3	36	112	13	8	58	4	39	102	9	15
135	0	9	98	139	0	1	583	2	50	88	10	6	40	3	31	76	6	28
136	0	9	102	218	0	2	527	3	114	94	16	6	36	5	47	137	11	28
137	0	9	103	229	0	2	622	4	27	82	14	5	53	4	44	149	10	33
138	0	9	104	155	0	7	486	4	12	93	12	9	43	4	36	97	6	28
139	0	9	101	208	0	5	347	3	12	105	11		98	3	32	102	5	38
140		9	99	509	0	2	499	2	65	159	14	9	94	4	41	415	9	24
141	0	8	102	195	1	2	600	2	21	178	14	15	43	4	44	82	10	25
142	0	9	97	115	1	1	419	4	33	91	12	19	38	4	36	62	8	23
143	0	9	92	208	0	3	273	3	22	85	15	8	93	5	45	99	6	38
144	0	9	105	174	0	1	365	4	21	101	12	12	57	4	39	96	6	30
145	0	9	103	253	1	2	331	3	15	175	15	15	41	4	44	184	5	32
146	1	9	102	172	0	2	458	3	28	97	14	6	44	5	44	113	7	21
147	0	9	104	201	0	3	310	3	10	95	10	7	61	4	34	113	5	31
148	0	9	103	211	0	2	370	3	23	165	13	16	65	4	41	113	6	22
149	0	10	105	241	0	2	374	3	44	98	14	6	68	5	45	151	5	46
150	1	9	101	165	0	2	188	2	55	107	16	12	33	5	46	112	5	34
151	1	9	103	205	0	2	335	4	11	233	12	4	43	4	38	121	9	32
152	0	9	99	185	0	1	316	4	18	112	12	10	41	4	35	128	5	32
153		9	102	185	0	2		4	22	96	15	10	55	5	47	85	7	17
154	1	9	107	190	0	6	352	2	15	96	14	118	47	3	40	126	4	31
155	0	8	103	217	0	1	398	3	8	109	12	6	57	4	35	129	6	32
156	0	8	100	161	0	4	625	4	12	96	9	8	50	3	29	77	10	22
157	0	9	99	233	0	1	296	3	9	93	13	4	54	4	39	162	4	32
158	0	9	104	207	1	3	373	3	40	93	16	11	52	5	47	134	6	27
159	0	9	105	164	0	1	383	2	20	113	13	3	53	4	39	98	6	19
160	0	9	99	192	1	2		4	122	95	14	28	53	4	42	111	12	41
161	1	9	106	180	1	2	274	3	19	94	16	5	73	5	46	95	4	41
162	0	9	103	165	0	2	319	3	12	101	12	3	74	4	37	74	5	30
163	0	10	101	237	1	1	265	3	83	99	17	5	75	5	50	117	5	23
164	0	9	99	155	0	6	527	3	137	85	12	6	42	5	38	97	6	27
165	1	9	104	205	0	4	260	3	32	104	13	4	55	4	40	132	5	30
166	0	8	107	195	1	0	321	2	22	117	16	11	38	5	48	123	8	25
167	0	9	100	177	0	2	264	2	38	108	14	5	52	5	41	103	5	31
168	0	10	103	189	0	2	313	3	21	169	13	12	49	5	41	96	6	36
169	0	9	98	180	1	2	412	4	44	130	11		43	4	32	106	8	15
170	0	9	109	217	0	3		3	13	81	13	28	68	4	42	125	4	48
171	0	9	105	255	1	1	345	3	15	117	14		38	4	40	171	7	32
172	0	9	105	221	0	3	361	4	11	102	14	4	97	4	43	114	6	39
173	0	9	102	179	0	2	236	3	13	101	13	5	54	4	39	98	6	60
174	0	9	101	175	0	1	393	5	36	160	11		71	4	34	90	7	30
175	0	8	111	227	0	1	330	3	18	84	13	3	49	5	39	141	5	34
176	0	10	97	192	1	1	393	3	48	211	14	21	48	5	42	119	8	25
177	0	9	101	209	1	4	338	4	65	99	15	7	48	5	44	110	8	25
178	0	9	104	147	3	3	557	4	55	101	8	15	45	3	27	69	7	15
179			101			3	415				14			4	41	0	6	33
180	0	9	100	241	0	1	380	4	28	115	14	8	48	5	44	156	6	38
181		8	91	164	0	2	855	3	132	86	11	9	43	3	35	106	10	5
182		9	91	226	1	2	793	4	59	134	7	8	45	2	24	144	6	21
183	0	9	106	152	1	2		4	23	73	15	10	57	5	44	75	6	27
184	0	9	101	201	0	1	254	5	9	90	10	9	70	4	35	105	8	24
185	0	11	103	249	0	2	407	4	33	114	13		55	4	38	171	9	32
186	0	9	98	187	1	1	443	3	21	108	14	11	49	5	41	108	8	30
187	0	10	99	177	1	4	406	3	28	110	16	9	41	5	46	121	7	31
188	0	9	107	196	0	5	316	3	41	105	15	9	42	4	43	93	8	30

Dataset. Relationship between Urinary Level of Phytate and Valvular Calcification in an Elderly Population: a Cross-Sectional Study

Subject	neutrophil_lymphocyte_ratio	LPA	Magnesium	Monocytes	Neutrophils	Potasium	Total protein	PTHi	Sodium	Triglycerides	Urea	Uric	VSG	MAC_ 1yes_ 2not	VAC_1 no or mild_2 mod or sever	terciles_ phytate
1	2,23	37	2	11	58	4	77	45	138	94	120	8	54	1	2	1
2	3,09	20	1	10	68	4	73	214	138	122	135	5	100	2	1	1
3	4,93	4	1	8	74	4	62	29	142	71	34	4	7	1	2	1
4	1,56	17	1	10	53	4	77	58	141	86	42	6	9	2	1	1
5	1,34	33	2	9	51	4	81	36	137	151	44	5	31	2	1	1
6	4,18	43	2	7	71	4	64	48	139	66	30	8	5	2	1	1
7	3,1	25	2	8	65	4	74	56	137	115	78	6	2	1	1	1
8	4,56	8	1	7	73	4	61	56	135	166	93	8	104	1	1	1
9	2,28	65	2	8	57	5	66	98	141	112	140	9	14	1	1	1
10	3,05	19	2	10	64	5	59	36	141	67	41	3	23	1	1	1
11	1,72	85	1	6	55	3	72	84	142	164	26	4	11	2	1	1
12	4,13	58	2	11	66	5	78	60	140	140	102	7	34	2	2	1
13	1,7	35	2	7	56	4	70	42	141	62	52	5	2	2	2	1
14	2,79	16	2	5	67	5	69	39	139	139	35	7	8	2	1	1
15	1,76	26	2	5	58	4	71	46	141	136	20	4	12	1	1	1
16	2,58	162	2	8	62	4	83	122	143	239	47	2	56	2	1	1
17	6,23	7	2	4	81	4	63	236	139	69	82	8	21	2	1	1
18	3,89	22	2	6	70	4	69	101	142	277	92	10	54	1	2	1
19	1,44	131	2	10	52	3	60	30	142	112	31	3	50	1	2	1
20	4,69	19	1	5	75	4	68	49	143	115	57	5	11	2	2	1
21	2,07	32	1	7	60	4	72	62	140	131	27	5	20	2	1	1
22	1,57	14	1	9	55	4	66	37	143	161	59	6	9	2	1	1
23	1,15	72	2	9	46	4	75	44	140	104	40	5	3	2	2	1
24	0,59	158	2	7	33	5	84	121	141	159	113	10	32	1	2	1
25	1,67	107	2	7	55	4	77	56	143	225	22	5	21	2	1	1
26	1,59	117	1	8	54	4	74	34	143	163	41	9	39	2	2	1
27	2,14	14	1	7	60	5	76	24	140	130	58	6	25	1	2	1
28	3,4	224	1	8	68	4	72	29	141	114	57	7	11	1	1	1
29	0	82	1	12	0	4	57	10	142	112	37	6	29	1	2	1
30	1,02	36	1	7	43	4	74	28	143	71	48	4	14	2	1	1
31	9,22	66	1	6	83	4	61	51	134	202	98	10	24	2	1	1
32	1,83	12	2	9	55	4	74	32	144	215	48	9	9	1	2	1
33	2,14	26	1	8	60	4	72	101	140	137	58	6	12	2	1	1
34	2,11	9	2	9	59	4	65	34	140	99	64	8	54	2	1	1
35	3,24	2	1	6	68	4	63	58	139	165	60	6	64	1	2	1
36	1,42	44	2	9	51	4	75	40	145	63	35	3	14	1	1	1
37	2,81	18	2	8	59	4	69	27	142	136	40	6	12	1	2	1
38	1,53		1	12	52	4	67	66	139	121	69	7	7	2	1	1
39	1	58	1	12	43	4	58	51	143	117	36	6	100	2	2	1
40	2,26	21	2	6	61	4	78	149	142	423	120	9	15	2	2	1
41	3,5	18	2	6	70	4	84	30	136	174	124	11	63	1	1	1
42	2,42	16	1	6	63	4	73	34	144	67	38	5	17	1	1	1
43	2,03	125	1	6	61	4	72	56	139	102	49	4	11	1	1	1
44	1,78	73	2	7	57	4	69	42	135	175	41	6	35	2	1	1
45	1,22	15	1	6	45	4	79	28	141	232	45	4	37	2	1	1
46	1,62	7	1	7	55	4	66	40	142	124	32	4	6	2	1	1
47	1,47	55	2	7	53	4	68	102	147	73	32	4	6	2	1	1
48	9,44	157	1	3	85	3	41	208	142	110	123	7	152	1	1	1
49	2,33	89	1	10	63	4	72	56	141	58	28	3		2	1	1
50	2,3	49	1	6	62		61	51	139	277	50	5	6	1	2	1
51	1,84	270	1	7	57	4	68	50	141	228	64	12	106	1	2	1
52	1,72	6	2	10	55	4	71	59	142	108	41	6	6	2	1	1
53	1,87	136	2	7	56	5	70	24	142	121	60	3	20	1	2	1
54	2,74	98	2	9	63	4	69	43	142	88	37	5	18	2	1	1
55	5,5	107	1	8	77	4	81	137	144	211	54	7	43	2	1	1
56	2,29		2	10	64	4	66	38	144	62	42	5	8	2	1	1
57	1,38	57	2	6	51	4	70	47	143	138	32	5	3	2	1	1
58	2,56	25	2	7	64	5	80	35	143	214	48	3		2	1	1
59	1,26	108	2	8	49	4	77	50	143	158	93	4	48	2	1	1
60	2,67		1	7	64	4	67	56	139	81	30	7	14	1	2	1
61	1,12	115	2	7	46	4	76	59	143	120	68	8	39	2	1	1
62	2,33	16	2	8	63	4	70	41	142	101	30	7	6	2	1	1
63	1,87	126	2	9	56	4	74	85	142	51	40	4	19	2	1	1
64	1,93	41	2	9	56	4	75	55	139	126	66	4	41	2	1	2
65	1,5	27	2	11	51	4	74	31	145	68	51	3	2	2	1	2
66	1,35	51	2	8	50	4	68	83	140	87	66	4	3	2	2	2
67	1,43	19	2	10	50	4	77	147	142	148	94	9	34	2	1	2
68	5,57	44	2	4	78	5	61	53	143	110	24	2	74	1	2	2
69	0,87	6	2	8	40	3	72	44	144	122	31	7	7	2	1	2
70	2,56	25	2	12	64	5	74	50	143	83	46	7	11	2	1	2
71	1,77	20	1	7	55	4	71	56	144	93	30	3	3	2	1	2
72	1,59	101	1	8	51	4	66	38	144	99	54	7	33	1	2	2
73	1,36	71	1	10	49	4	68	18	141	175	48	5	9	1		2
74	2,65	54	2	7	61	3	74	81	145	361	49	12	35	2	1	2
75	2,11	31	1	9	57	4	68	64	146	108	37	5	11	2	1	2
76	1,39	78	1	8	53	4	73	65	144	53	28	5	10	1	2	2
77	2		2	6	56	5	69	37	146	62	67	6		2	2	2
78	1,62	6	1	6	55	4	71	29	142	210	26	7	5	1	2	2
79	1,49	4	2	8	52	5	74	49	139	127	46	6	48	1	1	2
80	2,42			7	63	5			145				17	1	1	2
81	2,78	18	2	7	64	4	75	36	139	265	30	6	2	2	2	2
82	2,07	30	2	7	60	4	68	33	143	90	42	6	20	2	1	2
83	5,2	70	2	5	78	4	70	29	140	123	46	4	2	1	2	2
84	3,23	7	2	5	71	4	68	50	138	143	46	4	28	2	1	2
85	3,29	36	2	6	69	4	66	40	145	96	21	5	3	2	1	2
86	2,3	17	2	8	62	4	78	82	141	74	47	5	24	1	2	2
87	1,41	43	2	11	48	4	72	94	144	115	88	5	37	1	2	2
88	2,03	30	2	7	59	4	76	35	140	79	49	4	12	2	1	2
89	1,62	91	1	5	55	4	77	46	143	117	28	5	10	2	1	2
90	9,22	29	1	7	83	4	69	53	139	108	41	7	49	2	2	2
91	2,03	8	2	6	61	4	78	56	142	39	26	5	4	2	1	2
92	2,64	132	1	7	66	4	66	20	141	169	30	7	5	1	2	2
93	2,7	79	2	7	62	4	74	35	141	158	52	4	10	2	1	2
94	6,58	19	2	7	79	3	72	51	143	58	41	4	31	1	2	2
95	2,11	28	2	7	59	4	71	77	144	75	27	4	20	1	2	2

Subject	neutrophil_lymphocyte_ratio	LPA	Magnesium	Monocytes	Neutrophils	Potasium	Total protein	PTHi	Sodium	Triglycerides	Urea	Uric	VSG	MAC_ 1yes_ 2not	VAC_1 no or mild_2 mod or sever	terciles_ phytate
96	1,9	59	1	8	57	4	73	30	138	120	41	5	12	1	1	2
97	4,06	8	2	14	73	4	81	146	143	100	41	7	24	1	2	2
98	2,46	112	1	7	59	4	70	69	137	75	52	8	4	2	2	2
99	12,57	46	2	4	88	4	49	47	145	116	92	9	31	2	1	2
100	2,14	7	1	7	62	4	71	41	142	167	54	5	2	2	1	2
101	2,56	42	2	7	64	4	75	71	138	79	50	6	21	2	1	2
102	2,38	6	1	7	62	4	74	84	143	111	42	7	2	1	1	2
103	1,59	34	2	10	51	3	83	37	140	117	35	2	29	2	1	2
104	1,8	17	2	10	54	4	71	30	134	321	75	10	70	1	1	2
105	2,44	81	1	9	66	3	72	66	141	96	47	5	15	2	1	2
106	1,26	30	2	5	48	4	81	24	143	448	39	12	33	1	2	2
107	1,88	87	1	5	60	4	68	137	143	208	38	6	32	2	1	2
108	2,18	81	1	11	61	3	70	22	140	119	53	6	10	2	1	2
109	1,4	52	1	6	49	3	68	111	142	168	42	10	2	2	1	2
110	1,41	38	1	8	52	4	69	32	145	46	32	4	11	1	2	2
111	4,69	69	2	7	75	4	67	159	147	72	86	8	13	2	1	2
112	1,97	26	2	6	59	4	74	91	143	106	36	6	2	2	1	2
113	2,21	106	1	8	62	4	70	67	144	92	31	5	18	2	1	2
114	2,03	121	1	8	59	4	75	22	143	66	26	5	4	2	2	2
115	1,38	77	2	6	51	4	70	67	143	342	52	6	8	2	1	2
116	1,56	35	2	8	53	4	71	73	143	159	36	4	10	2	1	2
117	5	71	3	6	75	5	62	77	141	89	99	8	67	1		2
118	0,98		2	8	44	4	72	51	139	97	52	6	15	1	1	2
119	2,14	27	2	8	60	4	70	65	145	76	48	3	5	2	1	2
120	1,67	51	1	8	55	4	75	46	142	116	41	5	15	2	1	2
121	1,58	36	2	5	52	5	79	27	137	100	30	4	34	2	1	2
122	2,83	18	1	8	68	4	74	55	141	129	39	4	16	1	2	2
123	1,89	27	1	11	53	4	45	57	143	57	35	4	2	2	1	2
124	1,78	9	1	8	57	4	73	21	141	299	45	5	30	2	1	2
125	2,91	71	2	6	67	3	70	40	149	67	36	5	3	2	1	2
126	2,37	8	2	5	64	4	73	80	142	132	22	3	14	1	2	3
127	1,96	6	2	15	55	4	73	46	142	77	23	5		1	1	3
128	1,25	13	2	8	50	4	62	86	144	109	31	6	1	2	1	3
129	2,67	152	2	8	64	4	73	56	144	95	53	5	11	1	2	3
130	1,58	6	2	9	52	3	74	65	145	82	31	5	15	2	1	3
131	3,29	112	2	5	69	5	68	134	137	196	286	9	122	1	2	3
132	6,23	11	2	6	81	4	75	24	141	50	30	7	4	2	2	3
133	1,02	108	2	8	46	4	76	71	143	145	34	4		2	1	3
134	4,93	96	2	7	74	4	67	75	145	126	49	7	30	1	2	3
135	2	49	1	12	56	4	76	27	142	113	39	6	92	2	1	3
136	2,25	25	1	7	63	4	72	31	143	224	24	6	8	2	1	3
137	1,7	26	2	5	56	5	65	36	145	132	39	5	32	2	1	3
138	1,89	149	1	5	53	4	63	25	144	71	47	5	28	2	1	3
139	1,34	114	2	11	51	4	78	25	141	38	35	4	21	2	1	3
140	2,79		2	6	67	4	65	51	140		37	5	34	2	2	3
141	2,68	20	2	9	67	4	78	120	142	348	73	4	41	2	1	3
142	2,83	187	2	4	65	4	73	55	138	75	92	11	42	1	1	3
143	1,21	52	1	10	46	4	64	28	136	76	61	6	2	2	2	3
144	1,93	6	1	8	58	4	72	54	143	105	30	3	8	2	1	3
145	1,66	58	1	10	53	4	82	35	141	140	39	4	13	2	1	3
146	3,19	11	2	10	67	4	69	67	144	71	50	5	12	2	1	3
147	1,81	24	2	8	56	4	65	105	143	132	47	4	11	2	1	3
148	3,09	145	1	8	68	4	71	67	146	164	68	7		1	2	3
149	0,91	40	1	6	42	4	74	66	143	109	49	5	9	2	1	3
150	1,68	13	2	6	57	3	73	76	141	98	40	4	1	1	1	3
151	1,78	66	1	7	57	4	70	17	144	204	45	6	14	1	1	3
152	1,88	37	2	10	60	4	78	77	140	80	45	6	15	1	2	3
153	0,41	6	1	6	7		79	56	141	223	34	6		2	1	3
154	1,71	6	2	8	53	5	66	82	144	85	18	2	17	2	1	3
155	1,78	20	2	7	57	4	74	28	141	152	37	4		2	1	3
156	2,86	62	2	9	63	4	62	26	139	167	33	6	77	2	2	3
157	1,81	27	2	6	58	4	69	45	138	85	40	4	5	2	1	3
158	2,19	29	2	10	59	4	72	36	142	105	26	6	3	2	1	3
159	3,79	45	2	7	72	4	73	61	145	64	28	5	23	1	1	3
160	1,2	109	2	6	49	4	79	24	141	138	50	9	32	2	2	3
161	1,12	72	1	10	46	4	70	32	144	56	33	5	2	2	1	3
162	1,97	10	1	7	59	4	68	26	140	81	45	4	5	2	2	3
163	2,78	13	2	9	64	4	70	45	138	223	23	9	1	2	2	3
164	2,15	32	1	7	58	4	71	54	141	79	34	5	40	2	1	3
165	1,9	24	2	7	57	3	76	23	145	87	33	5	4	2	1	3
166	2,76	16	1	4	69	4	75	133	145	167	17	6	2	2	1	3
167	1,9	6	2	7	59	4	70	33	141	110	46	5	2	2	1	3
168	1,42	77	1	9	51	4	76	40	144	217	35	5	6	2	1	3
169	5	45	1	6	75	4	64	72	139	152	73	8	26	1	2	3
170	0,85	37	2	6	41	4	74	61	145	118	35	4		2	1	3
171	1,81	174	1	7	58	4	74	42	143	227	49	5	14	2	1	3
172	1,26	15	2	7	49	3	71	54	148	47	38	4	10	1	1	3
173	0,52	33	1	5	31	4	75	32	142	133	37	3	11	2	1	3
174	1,93	18	2	9	58	4	74	84	140	67	40	3	20	2	2	3
175	1,65	34	2	7	56	4	67	31	141	183	37	4	6	2	1	3
176	2,64	22	1	7	66	4	70	19	140	125	77	5	11	2	2	3
177	2,4	18	1	8	60	4	72	40	142	252	51	9	7	1	2	3
178	4,73	50	2	9	71	6	69	207	137	161	176	6	87	2	1	3
179	1,64			8	54	4			143				12	2	1	3
180	1,42	79	2	6	54	4	72	44	142	181	49	8	15	2	1	3
181	15,8	105	2	13	79		63	54	132	74	23	5	71	2	2	3
182	3,24	32	2	7	68		76	19	133	181	41	6	128	2	1	3
183	2,3	19	2	8	62	4	73	32	140	102	60	7		1	1	3
184	2,88	12	2	5	69	4	67	144	142	127	34	5	18	2	1	3
185	1,81	20	2	7	58	4	76	64	141	111	39	5	25	2	1	3
186	1,97	60	2	8	59	4	72	55	138	147	69	7	15	2	1	3
187	1,77	85	2	8	55	4	78	21	140	74	32	6	5	2	1	3
188	1,83	22	2	8	55	4	73	60	142	303	39	6	15	2	1	3
